# Atrial Natriuretic Peptide Antibody-Functionalised, PEGylated Multiwalled Carbon Nanotubes for Targeted Ischemic Stroke Intervention

**DOI:** 10.3390/pharmaceutics13091357

**Published:** 2021-08-28

**Authors:** Patrick P. Komane, Pradeep Kumar, Yahya E. Choonara

**Affiliations:** 1 Wits Advanced Drug Delivery Platform Research Unit, Department of Pharmacy and Pharmacology, School of Therapeutic Sciences, Faculty of Health Sciences, University of the Witwatersrand, 7 York Road, Johannesburg 2193, South Africa; patrickk@uj.ac.za (P.P.K.); pradeep.kumar@wits.ac.za (P.K.); 2 Department of Chemical Sciences, University of Johannesburg, 27 Nind Street, Johannesburg 2028, South Africa

**Keywords:** PEGylated carbon nanotubes, common carotid artery occlusion, multiwalled carbon nanotubes, atrial natriuretic peptide, brain ischemic stroke, dexamethasone

## Abstract

Stroke is one of the major causes of disability and the second major cause of death around the globe. There is a dire need for an ultrasensitive detection tool and an effective and efficient therapeutic system for both detection and treatment of stroke at its infancy stage. Carbon nanotubes are promising nanomaterials for tackling these challenges. The loading of dexamethasone and decoration of PEGylated multiwalled carbon nanotube with atrial natriuretic peptide (ANP) antibody and fluorescein isothiocyanate for targeting ischemic site in the rat stroke model is presented here. Functionalisation of carbon nanotubes with dexamethasone (DEX), polyethylene glycol (PEG), fluorescein isothiocyanate (FITC), and ANP antibody caused a 63-fold increase in the D band intensity as illustrated by Raman. The characteristic band intensity increase was observed at 1636 nm following functionalisation of carbon nanotubes with polyethylene glycol and dexamethasone as confirmed by Fourier Transform Infrared. These findings have demonstrated the coupling capability of atrial natriuretic peptide antibody to DEX-PEG-CNTs. The baseline plasma atrial natriuretic peptide levels were ranging from 118 to 135.70 pg/mL prior to surgery and from 522.09 to 552.37 following common carotid artery occlusion. A decrease in atrial natriuretic peptide levels to 307.77 was observed when the rats were treated with FITC-DEX-PEG-ANP-CNTs, PEG-CNTs and DEX with a significant drop in the FITC-DEX-PEG-ANP-CNTs treated group. Fluorescence was detected in FITC-DEX-PEG-CNTs and FITC-DEX-PEG-ANP-CNTs treated ischemic stroke rats. The highest fluorescence intensity was reported in plasma (2179) followed by the kidney (1563) and liver (1507). These findings suggest a beneficial role that is played by the FITC-DEX-PEG-ANP-CNTs in the reduction of inflammation in the ischemic stroke induced rats that could induce a successful treatment of ischemic stroke.

## 1. Introduction

Stroke is the root cause of disability and the second major cause of death globally as stated by the World Health Organization. About 70% of strokes are ischemic and the rest are intracerebral or subarachnoid haemorrhagic [1]. Ischemic stroke is a current threat to human health but there are limited therapeutic options due to the blood–brain barrier challenges [2]. There is an urgent need for effective and efficient diagnostic and therapeutic tools to overcome the current detection and treatment of stroke. Biomarkers are commonly used in the diagnosis of various diseases including stroke [3]. Atrial natriuretic peptide (ANP) and brain natriuretic peptide (BNP) are regarded as promising reliable biomarkers for a variety of medical conditions including ischemic stroke [4,5,6]. Diseases can be treated using a variety of techniques following proper diagnosis. 

Nanomaterials have been demonstrated to have potential in the treatment of a variety of diseases. Nanomaterials have advantages and disadvantages in their application in the field of medicine. Liposomes have high efficiency and low toxicity but they have a rapid elimination and inability to provide sustained drug release. Polymeric nanoparticles have increased the stability of highly volatile drugs but they are economically non-viable and their preparation process is complicated. Metal nanoparticles are free radical scavengers but they are toxic. Graphene has a higher surface area, but it lacks standardisation and is non-biogradable. Borophosphene is biodegradable but it is not an easy task to manipulate its shape and size. Hydrogels are also biodegradable and biocompatible but they are temperature sensitive and this may lead to excessive or insufficient drug release. Dendrimers have the ability to interact with charged functional groups but may have toxic effects [2]. In addition to nanomaterials, photothermal therapy has been found to be minimally invasive in the treatment of cancer by an ablation method using light irradiation in a clinically safe manner [7]. Black phosphorus photothermal therapy in combination with immunotherapy was employed by Xie and colleagues in the treatment of cancer in mice [8]. Borophene (Xene) has been regarded as a promising agent for energy, sensor, and biomedical applications [9]. Carbon nanotubes (CNTs) are promising nanocarriers for drugs to the point of interest for the treatment of diseases. They were discovered by Iijima in 1991 [10]. 

As compared to other nanomaterials involved in medicine, carbon nanotubes were demonstrated to be the most promising nanomaterials as they possess a unique one-dimensional structure with interesting intrinsic mechanical, physical, and chemical properties to cater for a plethora of applications in biology and medicine [11]. Carbon nanotubes are emerging smart nanomaterials that could be extremely useful in the development of technology as a result of their idiosyncratic anatomy and superlative physico-chemical characteristics [12,13]. CNTs have ultralow density and high specific surface area. They are non-immunogenic, non-toxic, biocompatible, photostable, cell membrane penetrable, and their size is alterable [14]. Carbon nanotubes possess three important unique features namely high specific surface area, ultralow density, and hollow central cavity for enabling the conjugation and encapsulation of therapeutics. Native CNTs are inert, hydrophobic in an aqueous environment, and toxic to cells as a result. Pristine carbon nanotubes agglomerate easily as a result of their inertness and van der Waals forces [15]. 

Functionalization plays a crucial role if carbon nanotubes are to be utilized in the biomedical field [16]. Functionalization makes carbon nanotubes compatible and stable in the biological milieu [17,18]. In addition, it allows carbon nanotubes to couple with a vast variety of biomolecules, diagnostic and therapeutic devices to generate accurate, economic and highly functional diagnostic and therapeutic tools [19]. Various promising data have so far been obtained about the application of carbon nanotubes in the treatment of diseases including cancer; however, the toxicity of pristine carbon nanotubes limits their application in a clinical setting.

The hollow tube-like structure of carbon nanotubes has the added advantages in drug delivery applications. This hollow inner cavity can hold molecules through adsorption and capillary action [20,21]. A spectrum of drugs can be encapsulated in their inner cavity, while other important molecules can be attached to the external walls to render them dispersible and biocompatible for targeting purposes. Based on this, the ANP antibody was used for targeting ischemic sites in the brain in stroke-induced rats. In this investigation, vertically aligned multiwalled carbon nanotubes were synthesized but their vertical alignment was lost during the purification and functionalization stages. Vertical alignment was performed to maintain the uniform length as carbon nanotubes with a variety of lengths have different effects on the cells [22]. Purification and functionalization affect the array as the carbon nanotubes are dispersed in different solvents to increase their specific surface areas to enhance loading capacity. These multiwalled carbon nanotubes were used to deliver ANP antibodies and dexamethasone to the ischemic site. Multiwalled carbon nanotubes were used as they possess improved physical and electrical properties for better interaction with other beneficial molecules.

Since pristine carbon nanotubes are inert in the aqueous milieu, they were purified and functionalized with various acids. They were also acylated to ensure higher reactivity and PEGylated to prevent them from being opsonized by macrophages and to avoid cleavage by some enzymes as they travel in circulation to their destiny. In healthy animals and humans, ANP levels are low but their expression increases when the animal is under stress. Furthermore, fluorescein isothiocyanate was also loaded for biodistribution studies to trace the location of the delivered drug.

In this study, the successful functionalisation of MWCNTs for application in theranostics of ischemic stroke was demonstrated. Furthermore, successful targeted delivery of dexamethasone to the ischemic site in Sprague Dawley rats that led to the reduction in symptoms of stroke was also demonstrated.

## 2. Materials and Methods

### 2.1. Reactions and Products of the Carbon Nanotube System

#### 2.1.1. Synthesis of Aligned MWCNTs and Morphological Evaluation

##### Aligned MWCNTs Synthesis

Vertically aligned multiwalled carbon nanotubes were produced by Chemical Vapour Deposition for use in preparing FITC-DEX-PEG-ANP-CNTs formulations as reported in the work of Komane and colleagues [23]. Silicon wafer (single side polished, <100>, N-type, contains No dopant, diam. × thickness 5 cm × 0.05 cm) from Sigma-Aldrich Corporation (St Louis, MO, USA) was cut into 1 cm^2^ pieces. The pieces were sonicated in acetone, methanol and water for the duration of 5 min. The silicon wafer was loaded into the quartz reactor tube to act as a platform for MWCNTs growth. The quartz tube was then inserted into a tube furnace (Elite TSH 12/38/500-2216E, Leicestershire, UK). The reaction vessel was activated with 40 mL of 25 mg/mL ferrocene. A 95% baseline argon balanced with 5% hydrogen was used as a carrier gas. The flow rate of the argon-hydrogen mixture was set to 50 mL/min to create an inert environment using a mass flow controller (D08-4D/ZM, Beijing Sevenstar, Huachuang Electronic Co., Ltd., Beijing, China). The nebulizer (Dr Hielscher UM20-1.6 MHz Sonic, Teltow, Germany) was used to generate an aerosol. The synthesis temperature was set to 775 °C at a ramp of 10 °C/min, gas flow rate to 400 mL/min, and synthesis time to 45 min.

##### Morphological Evaluation of the CNTs

SEM was performed to determine the orientation of the CNTs (aligned or entangled) and the length of the CNTs. As-synthesized CNTs were collected from the quartz tube and prepared for SEM analyses in order to study their morphology and size. CNTs were placed on a carbon tape or aluminum tape on the stub and coated with Pd/Au using Emitech K550X (Emitech Ltd., Canterbury, Kent, UK) for 4 min at 25 mA and 2 × 10^−1^ mbar. CNTs samples were then loaded on FEI Nova NanoLab 600 FEG-SEM/FIB (FEI Company, Hillsboro, OR, USA) for morphological evaluation. 

TEM analysis was performed to determine the type of CNTs (single or multiwalled CNTs) and the size (internal and external diameters). A small amount of CNTs was transferred into a 1.5 mL Eppendorf tube and dispersed in absolute ethanol by ultrasonication in an ultrasonic water bath (Materials, Inc., Danbury, CT, USA) for 10–15 min. The Cu grid was placed on a Whatman filter paper and Pasteur pipette was used to transfer dispersed CNTs onto a Cu grid. The grid with the loaded CNTs was dried under light using a light microscope. The sample-loaded Cu grids were loaded on FEI Tecnai T12 TEM (Hillsboro, OR, USA) operating at 120 kV for the determination of external and internal diameters of CNTs. ImageJ software was used to measure the length and diameters of the CNTs.

#### 2.1.2. Purification, Acid Functionalization, Acylation and PEGylation of MWCNTs

##### Purification of the MWCNTs

**Chart 1:**
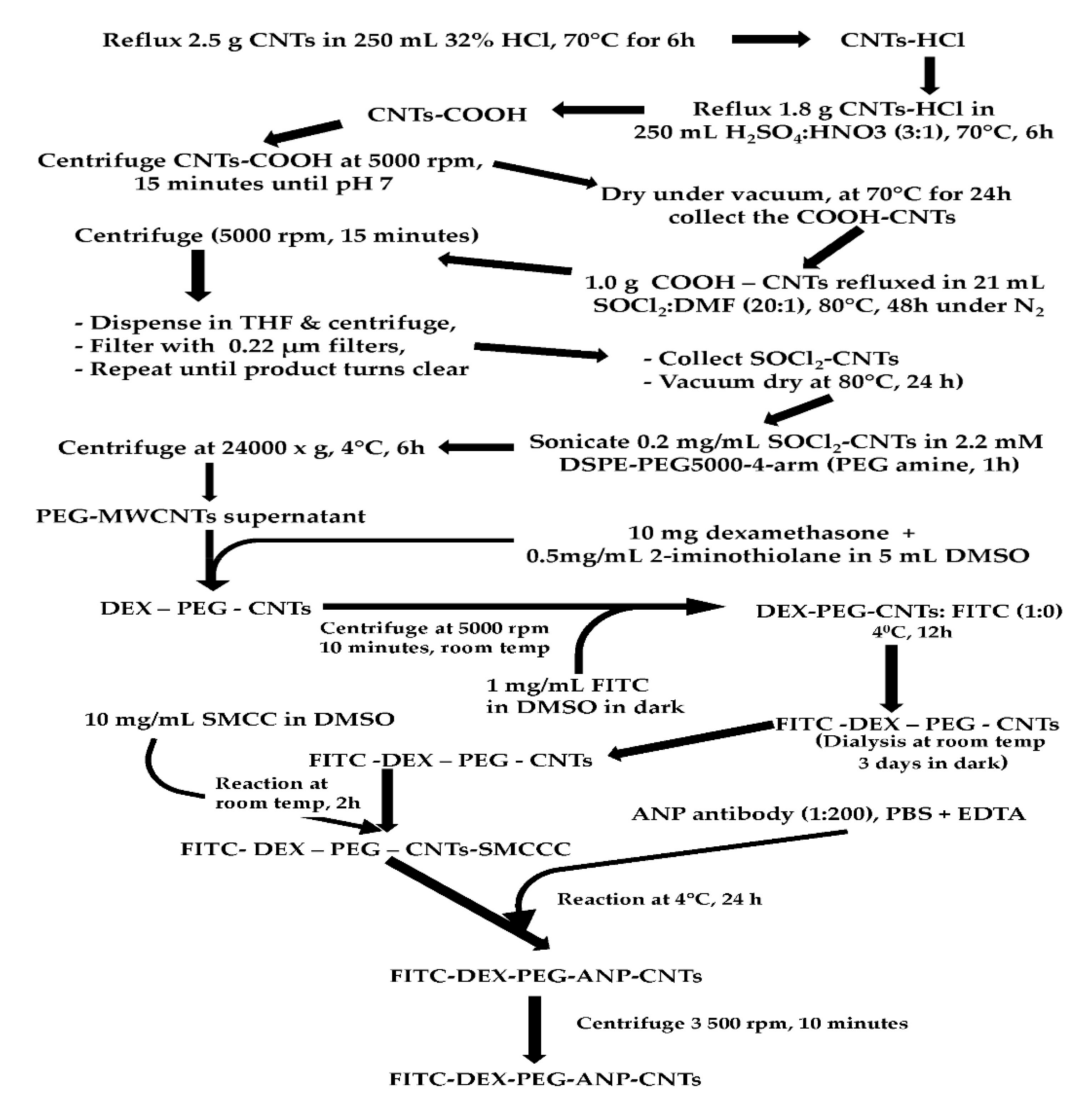
Steps for the production of FITC-DEX-PEG-ANP-CNTs.

An amount of 2.5 g of MWCNTs was purified by refluxing in 250 mL of 32% HCl in a round bottom flask at 70 °C for 6 h (Chart 1). 

##### Carboxylation of the Purified MWCNTs

An amount of 1.8 g of HCl-purified MWCNTs was refluxed at 70 °C for 6 h in H_2_SO_4_:HNO_3_ (3:1). MWCNTs were then washed through centrifugation until the pH was neutral. They were dried under vacuum at 70 °C for 24 h to eliminate water from the carbon nanotubes (Figure S1A). Carboxylated MWCNTs were collected for characterization and further functionalization (Chart 1). 

##### Acylation of the Carboxylated MWCNTs

Following carboxylation, MWCNTs were acylated with SOCl_2_. An amount of 1.0 g of MWCNT-COOH was added to 21.0 mL of SOCl_2_:DMF (20:1) mixture. The solution was refluxed for 48 h at 80 °C under nitrogen (Chart 1).

The product was centrifuged at 5000 rpm for 15 min. Both the supernatant and precipitate were washed with THF to remove SOCl_2_ using filtration via a 0.22 µm filter and centrifugation, respectively until the brown color due to SOCl_2_ vanished [21]. The SOCl_2_-MWCNTs were collected from the filters and centrifuge tubes and dried at 80 °C for 24 h using a dry vacuum oven (Trade Raypa, Terrassa, Spain) to remove water without interfering with the chemical properties of the complex (Chart 1).

##### PEGylation of the Acylated MWCNTs

Acylated MWCNTs (0.2 mg/mL) were sonicated with 0.2 mM DSPE PEG5000-4-arm (PEG-amine) for 1 h (Chart 1). The product was centrifuged at 24,000× *g* for 6 h using a Centrofriger-BL11 centrifuge (JP-Selecta, Abrera, Barcelona, Spain), and the supernatant was collected for characterization and further use [14]. 

#### 2.1.3. Dexamethasone Loading to PEG-CNTs, FITC Labelling and ANP Antibody Conjugation to FITC Labelled PEG-CNTs

##### Loading of Dexamethasone to PEG-CNTs

Dexamethasone (50 µg/mL) was thiolated to add amino group using 2-iminothiolane in dimethylsulfoxide. Thiolated dexamethasone was reacted with DSPE-PEG5000-4-arm (PEG-amine) in a 5:1 ratio for 24 h at room temperature to generate DEX-PEG-CNTs. DEX-PEG-CNTs were centrifuged at 5000 rpm for 10 min and the supernatant was stored at 4 °C for future use (Chart 1). 

##### FITC Labelling of DEX-PEG-CNTs

A fresh solution of 1 mg/mL of FITC was prepared by dissolving 1 mg of FITC in 1 mL of DMSO. This solution was then reacted with DEX-PEG-CNTs in a ratio of 1:10 at 4 °C for 12 h in the dark (Chart 1).

The mixture was dialyzed against fresh ultrapure water for 3 days to generate FITC labelled DEX-PEG-CNTs (Figure S1B). The unbound FITC was removed by passing through the dialysis membrane into the water leaving behind the FITC-DEX-PEG-CNTs in the dialysis tubing (Chart 1).

##### ANP Antibody Conjugation

A volume of 0.3 mL of 10 mg/mL succinimidyl-4-(*N*-maleimidomethyl) cyclohexane-1-carboxylate (SMCC) in DMSO was added to 5 mL of FITC-DEX-PEG-CNTs. SMCC is a heterobifunctional cross-linker that is used in bioconjugation to link proteins with other functional entities such as fluorescent tags, tracers, and nanoparticles [24]. SMCC was used in this study to conjugate the ANP antibody to FITC- DEX-PEG-CNTs. To this solution, 0.6 mL of 10 × PBS was added and the reaction was allowed to proceed for 2 h at room temperature. 

Approximately 100 µL of ANP antibody (1:200), 500 µL of 10 × PBS, and 20 µL of 0.5 M EDTA (Sigma, Cat. No.: 03690-100ML) were added and the mixture was incubated at 4 °C for 24 h. The reaction mixture was centrifuged in a filter device with MWCO of 100 kDa at 3500 rpm for 10 min to remove unbound ANP antibody and SMCC and the supernatant was collected for characterization and drug release studies.

#### 2.1.4. FTIR Spectroscopy of MWCNTs

KBr was mixed with CNTs and finely ground and loaded on KBr die. The mixture was pressed at 4-ton pressure under vacuum for 15 min with KBr press (ICL, International Crystal Labs, Garfield, NJ, USA) to produce a circular KBr pellet. The Perkin Elmer Spectrum 2000 FTIR spectrometer (PerkinElmer Spectrum 100, Llantrisant, Wales, UK) was set at a resolution of 4 cm^−1^ and 10 accumulations at wavenumber from 4000–400 cm^−1^. For aqueous samples, ATR was used for the collection of the IR spectra.

#### 2.1.5. Raman Spectra of the Functionalised CNTs

A Raman Micro200 (Perkin Elmer, Waltham, MA, USA) was used to analyze the functionalized CNTs. A laser beam of 785 nm with a wavelength from 200 to 2500 cm^−1^ at 4 cm^−1^ resolution with a maximum output of 250 mW was used to collect the spectra. Samples were placed on quartz slides on the microscope stage and the focus was adjusted to obtain the best image using objective 50× and 20× under the bright field. Scanning was performed under the dark field.

#### 2.1.6. UV–Vis and Fluorescence Evaluation in the Functionalised CNTs

Functionalized CNTs were suspended in water and ultrasonicated prior to scanning. UV–Vis spectra were measured with a Shimadzu UV2450 spectrometer (Shimadzu Corporation, Kyoto, Japan) using a scanning range from 190 to 1100 nm at room temperature. 

Excitation and emission scans of the CNTs were collected by an LS45 Perkin Elmer fluorescence spectrometer (PerkinElmer, Beaconsfield, UK) with a scan range from 200 nm to 800 nm. Fluorescence was also determined by Aqualog FEEM spectrometer (Horiba Scientific, Piscataway, NJ, USA) in a 3D mode.

#### 2.1.7. Dexamethasone Release Studies

Dialysis of DEX-PEG-CNTs was performed against 200 mL of 0.1 M PBS buffer with pH 7.4, 6.5, and 5.5, respectively, in an orbital shaker (YIHDER, Xinbei City, Taiwan) at 37 °C with 30 rpm speed. Collection of 3 mL of the PBS buffer was carried out at various time intervals from 0 to 12 h (0 h, 0.5 h, 1 h, 2 h, 4 h, 6 h, 8 h, 10 h, 12 h). The sink conditions were maintained. The λmax of dexamethasone was determined using UV–VIS (Shimadzu Corporation, Kyoto, Japan) and was found to be 242 nm. The standard curve of dexamethasone was then constructed using a variety of concentrations and was used to obtain the levels of dexamethasone in 3 mL aliquots of PBS buffer collected at various time intervals during a 12 h dialysis of the DEX-PEG-CNTs.

### 2.2. Biological Properties of the Carbon Nanotube System

#### 2.2.1. Evaluation of Cytotoxicity of FITC-DEX-PEG-ANP-CNTs

PC-12 neuronal cells were used for evaluating the toxicity of the formulation prior to neuro-application. Cryopreserved PC-12 cells were grown in an incubator at 37 °C and 5% CO_2_ incubator in RPMI 1640 culture medium supplemented with glutamine, 5% fetal bovine serum (FBS), and 10% horse serum (HS). FBS and HS were inactivated by heating at 56 °C for 30 min in a water bath prior to introduction into the culture medium. A mixture of 1% penicillin and streptomycin (pen/strep) was added to the culture medium, and cells were checked on a daily basis for viability and microbial contamination by the Trypan blue exclusion technique.

PC-12 cells were seeded in 96-well plates at a concentration of 6 × 10^4^ cells/mL per well with FITC-DEX-PEG-ANP-CNTs ranging from 0 to 20 µg/mL in PBS (pH 7.4) for 2 h. Following incubation, an MTT assay was performed to evaluate the cytotoxic effect of the formulation at various concentrations. Samples were cultured in 96-well microplates and incubated with the MTT solution for approximately 4 h. Water-insoluble formazan dye was formed and solubilized by solubilization solution. The formazan dye was quantitated at 570 nm using Multilabel Reader (Victor X3, Perkin Elmer, Waltham, MA, USA).

#### 2.2.2. Animal Administration and Experimental Design

**Figure 1:**
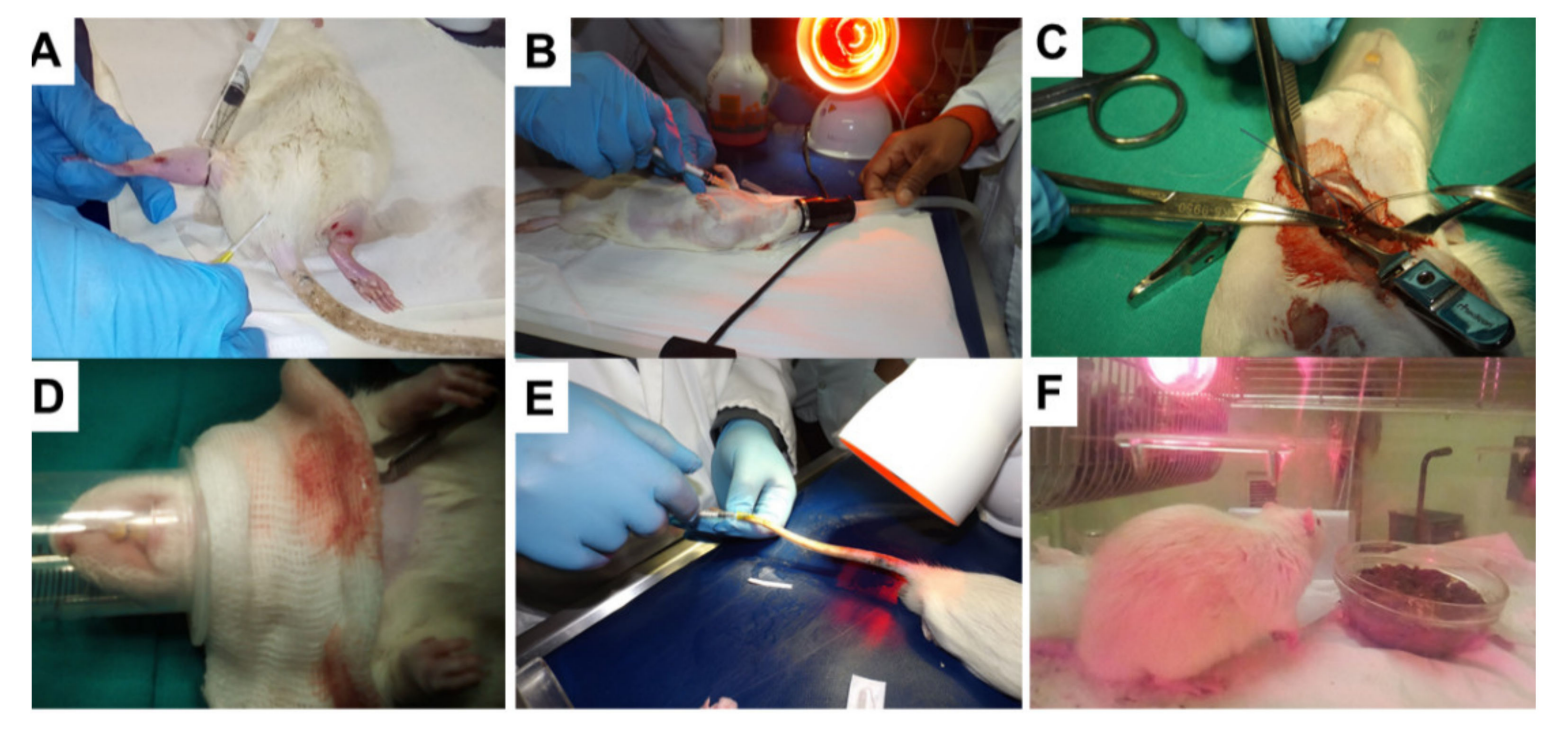
Unilateral common carotid artery occlusion. Blood withdrawal from the saphenous vein (**A**). Injection of local anaesthetic (lignocaine) on the neck (**B**). Occlusion of CCA by clamping (**C**). 30 min occlusion (**D**). Blood withdrawal from the tail vein (**E**). Recovering from anaesthesia (**F**).

Male Sprague Dawley rats (360–560 g) were used in in vivo studies and all protocols of the study were authorized by the Animal Research Ethics Committee (AREC) of the University of Witwatersrand Medical School (Ethics clearance no. AREC # 2017/03/17/D). Rats were kept in pairs in an enriched environment air-conditioned with a temperature of 26 °C. Rats were accommodated in a 12-h light/dark cycle environment with food and water ad libitum. They were acclimatized to a new environment for at least one week prior to surgery. Rats were haphazardly split into six categories according to the formulations. Unilateral common carotid artery occlusion (ULCCAO) was conducted to induce stroke as demonstrated in Figure 1.

#### 2.2.3. Unilateral Common Carotid Artery Occlusion (CCAO) and Neurobehavioural Assessment

Unilateral common carotid artery occlusion was conducted to induce stroke in rats. Rats were weighed a day before surgery to avoid or minimise stress and the weights were also used to determine the amount of the formulations to be injected into the rats. A neurobehavioural evaluation was conducted prior to surgery to establish a baseline.

A long-lasting analgesic (0.1 mL Temgesic) and inhalation anaesthesia (3% Isoflurane) were used to anesthetise the rat. A 0.2 mL of blood was collected from the tail vein or saphenous vein into the heparinised blood collection tube (Figure 1A). The neck was shaved and local anaesthetic (0.2 mL Lignocaine) was administered subcutaneously on the shaved area (Figure 1B). 

Anaesthesia was maintained under 1–2% Isoflurane and 200 mL/min O_2_ (Figure 1B). ULCCA was exposed and ligated with 3–0 silk sutures for 30 min (Figure 1C,D). Ligation was removed after 30 min, wound closed and a volume of about 0.2 mL blood was collected from the tail prior to surgery and immediately after surgery prior to full recovery (Figure 1E). 

#### 2.2.4. Optimization of the Formulation Dose 

This study was undertaken to ensure that the doses of the formulations that will be used in the main study were not toxic to the rats. ULCCAO was performed as in Section 2.2.3 and occlusion was removed, and all the incisions were sutured. Blood (0.2 mL) was collected into heparinized tubes. Dose–response studies were performed using 1, 10, and 100 µg/Kg body weight of dpcaf (functionalised carbon nanotubes) to determine the dose at which dpcaf was toxic to the rats. Rats were transferred to the recovery room (Figure 1F) and a neurobehavioural assessment was undertaken thrice within 24 h of reperfusion. The pellets were soaked in water to soften them for the rat to feed with ease following surgery (Figure 1E). 

#### 2.2.5. Treatment of Stroke Rats with the Functionalised Carbon Nanotubes

All carbon nanotube concentrations were found to be non-toxic to rats and the 100 µg/Kg was used in the subsequent injections. The 0.2 mL blood was transferred into the heparinized blood collection tube before and immediately after surgery. The neurobehavioural assessment was performed prior to surgery, immediately after surgery and within 24-h reperfusion at an interval of 4–6 h. Rats were strictly monitored to ensure that they do not suffer from post-surgery complications. They were checked every 4 h and injected with Temgesic every 6 h. They were then euthanized after 24 h. Blood was collected using a cardiac puncture technique and organs (brain, liver, heart, kidneys, spleen, and lungs) were collected for biodistribution studies. 

#### 2.2.6. Tissue Sample Preparation

Tissues were cut into smaller pieces of 0.2–0.5 g (balance Mettler AJ100, Zürich, Switzerland) and homogenised in 2–5 mL phosphate-buffered saline (PBS) using a glass homogenizer. They were further homogenised on ice at 10 s pulse, 60% amplitude using an ultrasonic processor (Sonics Ultracell, Newtown, CT, USA).

The homogenates were centrifuged at 12,000 rpm for 10 min in a microcentrifuge (TopScien, Ningbo, China). Supernatants were collected, and fluorescence intensities were determined using a microplate spectrophotometer (2030 Multilabel Reader Victor X3, Perkin Elmer, Waltham, MA, USA).

#### 2.2.7. Determination of Fluorescence Intensity in the Rat Tissues

The stock solution of 10 µg/mL of the functionalised carbon nanotubes was prepared by dispersing 1 mg carbon nanotubes in 100 mL PBS with pH 7.4. The concentrations (0.2, 0.6, 0.8, 1.4, 2, and 4 µg/mL) of the functionalised carbon nanotubes (FITC-DEX-PEG-ANP-CNTs) were prepared by serial dilution in PBS buffer, pH 7.4 from the stock solution. Black 96-well plates were used to avoid or reduce the auto-fluorescence from the samples. Each well of the 96-well plates were loaded with 100 µL of each concentration in triplicates. 

The supernatants from various tissues of the rat organs were also loaded in triplicates. Fluorescence intensity was measured at an excitation wavelength of 490 nm and an emission wavelength of 520 nm using a microplate spectrophotometer (Victor X3, Perkin Elmer, Waltham, MA, USA) equipped with a fluorescence filter with a wavelength of 520 nm. Fluorescence intensity was equivalent to the amount of the functionalised CNTs in different organs.

#### 2.2.8. Histological Evaluation of the Rat Brain Tissue

Following harvesting of the brains, the right and left hemispheres were separated, weighed and their volumes determined using deionised water in a volumetric flask. They were stored in 10% formalin for histological evaluation. Following washing with PBS and dehydration with ethanol, the specimens were embedded in wax blocks. They were sectioned into 5 μm sections using a microtome. They were stained with haematoxylin and eosin and mounted on the slides and viewed under an inverted microscope (Olympus CKX53, Tokyo, Japan). Since some specimens were treated with FITC, the slides were also viewed under fluorescence LSM 780 Confocal Microscope at 10× objective (Zeiss, Gottingen, Germany) equipped with a 488 nm laser.

#### 2.2.9. Determination of Plasma ANP Levels in the Rat Venous Blood

The competitive ELISA technique was used to measure the levels of plasma ANP. MyAssay program was used to determine the averages and SEM of the triplicates of the reference standards and samples. A 4-parameter logistic curve was plotted from the standards and the concentrations of ANP were determined from the curve. A rat ANP ELISA kit was used for the determination of plasma ANP levels in the non-ischemic untreated and ischemic treated rats. The ELISA kit operates on the competitive-ELISA principle. The micro ELISA plate with 96 wells was coated with Rat ANP prior to use. The sample or standard ANP competes with a fixed amount of rat ANP coated on the microplate wells for the sites on the Biotinylated Detection Antibody specific to rat ANP.

Surplus conjugate and free unbound samples and standards were washed away from the plate, and Avidin conjugated to Horseradish Peroxidase (HRP) was added to each well. The plate was incubated for a certain period of time at a specific temperature for the reaction to take place. A substrate was added to each well and the blue colour developed. The stop solution was added to terminate the enzyme-substrate reaction. The reaction mixture in each well changed to yellow. The absorbance of each well was determined by a spectrophotometer at a wavelength of 450 nm ± 2 nm.

The ANP concentration in the samples is determined by the extrapolation of the standard curve. In this study, the absorbances of ANP standards with concentrations ranging from 0–1000 pg/mL were recorded by a microplate reader at 450 nm. Absorbance was plotted against concentration and the concentrations of the unknowns were determined from this standard curve by extrapolation. A negative slope was generated as this is a competitive ELISA procedure. 

#### 2.2.10. Statistical Analysis

Male rats were used in this study to avoid the effect of oestrogen on inflammation from females. In this study, six groups with six rats per group were used and the groups were categorised as follow: PREMED, DEX, SHAM, DEX-PEG-CNTs, FITC-DEX-PEG-CNTs and FITC-DEX-PEG-ANP-CNTs. A Student’s t-test was employed to assess the statistical significance of the results. *p*-values < 0.05 were considered statistically significant. 

## 3. Results and Discussion

### 3.1. Reactions and Products of the Carbon Nanotube System

#### 3.1.1. SEM and TEM Morphological Evaluation

SEM and TEM techniques were employed to determine the morphology of carbon nanotubes that were synthesised using the Chemical Vapour Deposition technique.

**Figure 2:**
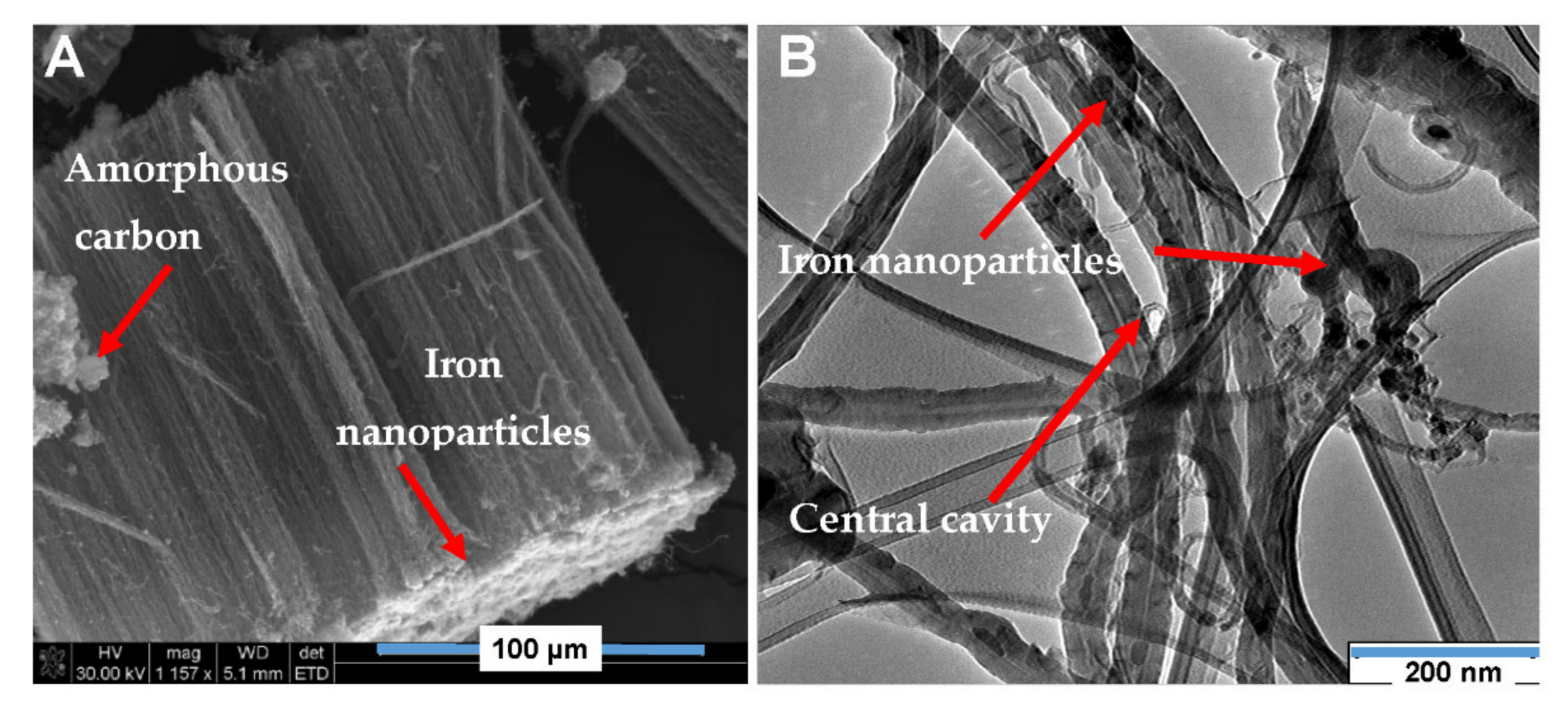
Vertically aligned multiwalled carbon nanotubes. SEM micrograph showing vertically aligned multiwalled carbon nanotubes with amorphous carbon and iron nanotubes (**A**). TEM micrograph with iron nanotubes embedded in the central cavities (**B**).

Vertically aligned multiwalled carbon nanotubes were produced with the following dimensions: 165 µm length, 11 nm internal diameter, and 46 nm external diameter as depicted in Figure 2A,B. CNTs were contaminated with iron nanoparticles and amorphous carbon during synthesis as demonstrated in Figure 2A,B (red arrows).

#### 3.1.2. Preparation of FITC-DEX-PEG-ANP-CNTs

**Scheme 1:**
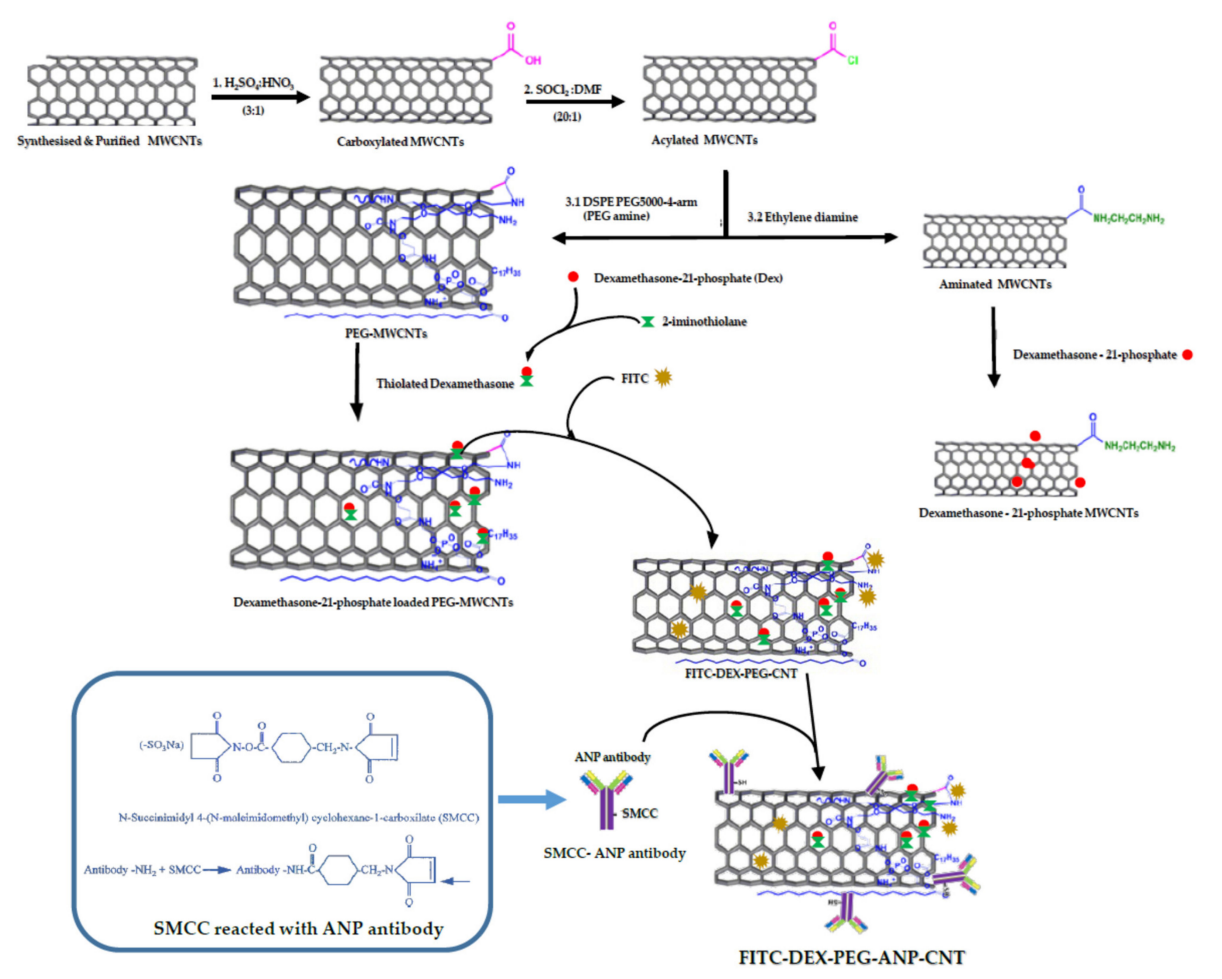
Schematic representation showing the preparation of FIX-DEX-PEG-ANP-CNTS.

Aligned MWCNTs were purified using HCl to remove contaminants such as iron particles from the catalyst (ferrocene) and the amorphous carbon that was synthesised. HCl acid removes Fe from the catalyst in the following manner: Fe (s) + 2HCl (aq) → FeCl_2_ + H_2_ (g). Purified CNTs were carboxylated by H_2_SO_4_:HNO_3_ (3:1) reflux to add reactive COOH functional groups on CNTs. The carboxylated CNTs were acylated to add more reactive acyl chloride groups on their walls by reacting them with SOCl_2_ (Scheme 1).

The CNTs were equipped with more reactive functional groups ready for further functionalisation. The acylated MWCNTs were PEGylated by reacting them with DSPE-PEG5000-4-arm (PEG-amine). The DSPE-PEG5000-4-arm (PEG-amine) was synthesised by reacting DSPE-PEG5000 amine with 4-arm (PEG- amine) [25] in order to increase the branches to make it more reactive (Scheme 1). Dexamethasone was thiolated using 2-iminothiolane to enhance its conjugation with PEGylated CNTs [14]. FITC was reacted with PEG-CNTs to attach it to the surface of the CNTs via π-π interactions and to conjugate it to the PEG-CNTs via PEG chains [18]. 

ANP antibody was reacted with SMCC to enable it to conjugate with PEG-CNTs (Scheme 1). SMCC is a heterobifunctional water-soluble amine to sulfhydryl cross-linker that is equipped with NHS ester and maleimide reactive groups at the opposite ends of the cyclohexane spacer arm. NHS esters react with primary amines at pH 7–9 to form stable amide bonds. Maleimides react with the sylfhydryl group at pH 6.5–7.5 to form stable thioester bonds for antibody–CNTs conjugation.

#### 3.1.3. Mechanism of ANP Targeting

ANP is an inflammatory biomarker that is up-regulated at the site of a stroke event in stroke patients [26]. The circulating biomarkers are distributed all over the body but the highest concentration is located at the site of inflammation (e.g., ischemic site) and hence the ANP antibody–drug complex predominantly accumulates at the diseased site as a result of the highest concentration of these biomarkers [27].

**Figure 3:**
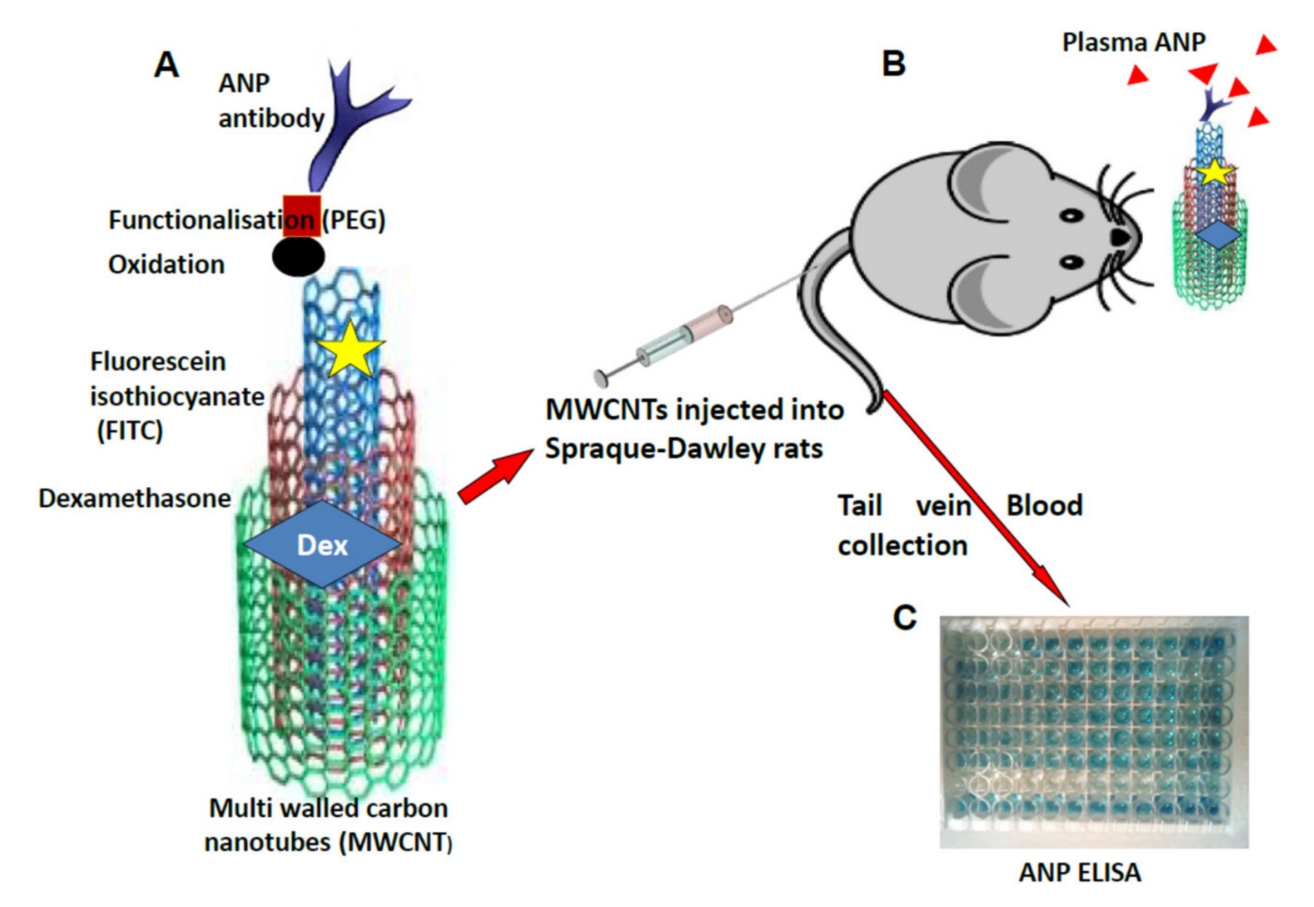
ANP mechanism of targeting the inflammatory site of stroke. (**A**) Functionalised MWCNT; (**B**) Rat injected with functionalised MWCNT; (**C**) Detection of ANP using ELISA technique.

An injectable carbon nanotube system (FITC-DEX-PEG-ANP-CNT) was designed for drugs targeting the diseased site. The system was equipped with the MWCNTs as nanocarriers, FITC as a tracer, dexamethasone as an anti-inflammatory drug, PEG as a multipurpose tool for conjugation and protection against opsonization, and ANP antibody as a targeting agent against plasma ANP (Figure 3A).

This carbon nanotube-based system is intravenously injected into the rat via a tail vein or saphenous vein (Figure 3B). Since the ANP are predominantly accumulated at the diseased site, the ANP antibodies preferentially bind to the biomarkers at the ischemic site in the brain and less so peripherally outside the brain or in the blood. Dexamethasone, an anti-inflammatory drug, gains access to the inflamed site and acts against inflammation. Since the BBB is leaking, therefore the ANP leaks into the blood circulation (Figure 3B). Blood is collected and plasma ANP is detected using the ELISA technique (Figure 3C).

#### 3.1.4. FTIR Assessment of the Functionalised MWCNTs

**Figure 4:**
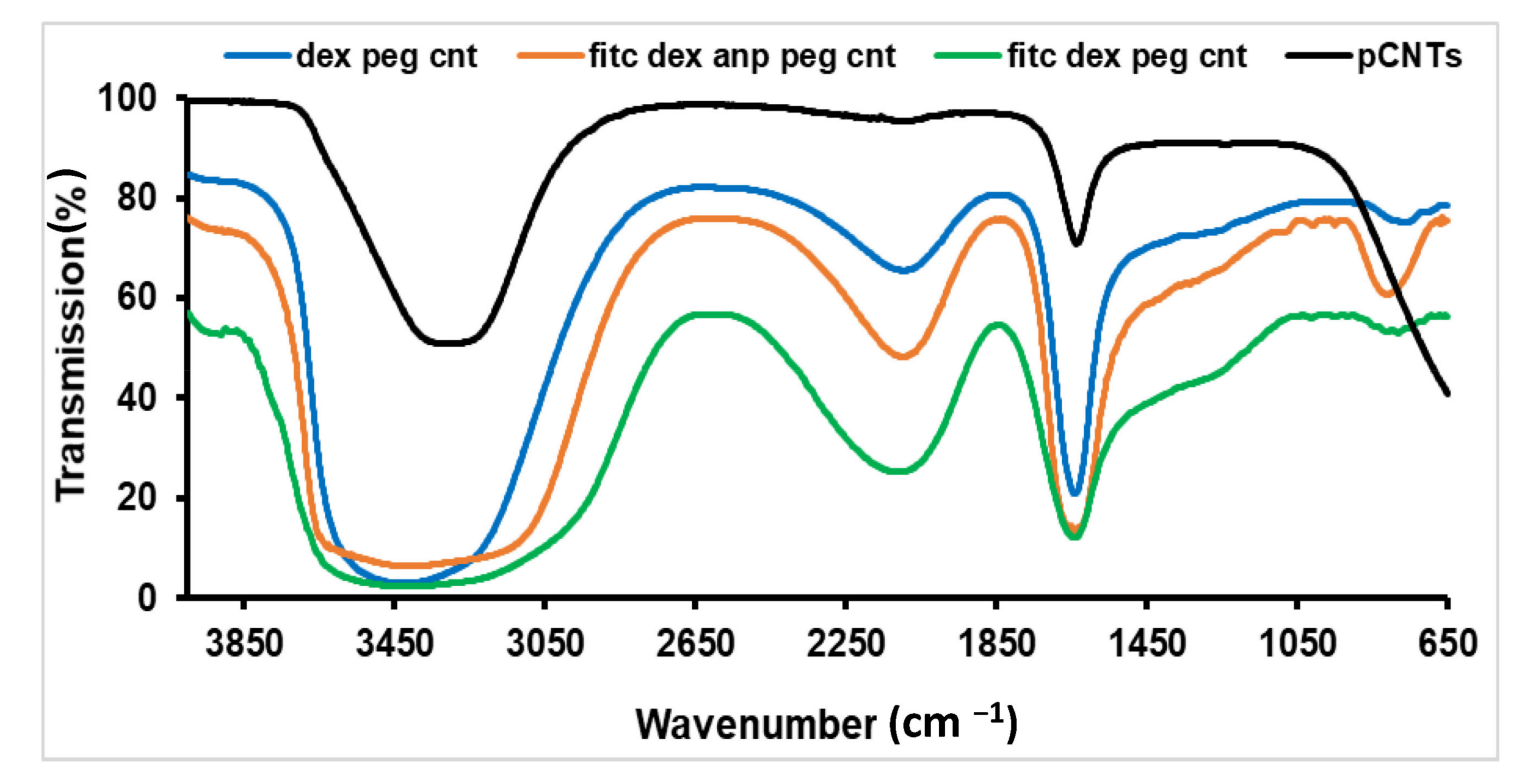
FTIR Spectra of the functionalised VA-MWCNTs. FTIR spectra of FITC-DEX-PEG-CNTs, FITC-DEX-PEG-ANP-CNTs and DEX-PEG-CNTs formulations in deionised water.

The optical properties of the pristine CNTs were impacted by functionalization with PEG, FITC, DEX, and ANP antibody as depicted in Figure 4. Two characteristic bands at 3410 nm (O–H) and 755 nm (C–H) were observed in pristine CNTs whereas four characteristic bands were observed at 3410 nm (O–H), 2086 nm (C≡C), 1636 nm (C=C) and 755 nm (C–H) in DEX-PEG-CNTs, FITC-DEX-PEG-CNTs, and FITC-DEX-PEG-ANP-CNTs. The characteristic band intensities increased in all the functionalised CNTs compared to pristine CNTs with a drastic increase at 755 nm and 1636 nm in FITC-DEX-PEG-ANP-CNTs as compared to DEX-PEG-CNTs and FITC-DEX-PEG-CNTs. The intensity of the characteristic band at 1636 nm in DEX-PEG-CNTs increased when FITC and ANP antibody were coupled to DEX-PEG-CNTs implying that the size of the functional groups (C=C) at 1636 nm was increased (more of the same group added). 

The intensity of the characteristic band at 755 nm in DEX-PEG-CNTs increased and became broader and shifted to the left to 799 nm as the ANP antibody was coupled to the complex. These changes implied that a new bond was formed due to the addition of the ANP antibody to DEX-PEG-CNTs. Only two characteristic bands (3250 nm and 1650 nm) with lower intensities compared to the rest were observed in pristine MWCNTs (Figure 4).

The characteristic band at 799 nm (C-H) in FITC-DEX-PEG-ANP-CNTs was very prominent compared to the rest. The characteristic band at 3410 nm had a higher intensity in DEX-PEG-CNTs. In FITC-DEX-PEG-CNTs, the characteristic band became weaker and broader as a result of the loading of FITC to DEX- PEG- CNTs. The intensity and strength of the FITC-DEX-PEG-CNTs at 3410 nm was higher than that of the DEX-PEG-CNTs and FITC-DEX-PEG-ANP-CNTs.

The characteristic band at 3410 nm was broader and most prominent as a result of the presence of water (OH group) that was used as a dispersant for dispersing the functionalised CNTs. A characteristic band at 2114 nm (C≡C) with high intensity and 790 nm (fingerprint) with very low intensity were observed in FITC-DEX-PEG-CNTs (Figure 4). 

In pristine multiwalled carbon nanotubes, the intensity of the major characteristic band was very low and had 60.5% transmission as there was no interaction of light with other molecules. The intensity of the major characteristic band of pristine multiwalled carbon nanotubes increased when carbon nanotubes were functionalised with SOCl_2_ and HNO_3_. There was also a reduction in transmission percentage to 43% for HNO_3_ treated and 37% for SOCl_2_ treated MWCNTs (Figure S2A).

An additional characteristic band was observed at 801 nm in SOCl_2_ functionalised MWCNTs. The reduction in transmission percentage and a rise in the intensity of the characteristic band confirmed the interaction of MWCNTs with the functional groups. The characteristic band at 801 nm in SOCl_2_ confirmed the C-H functional group as demonstrated in Figure S2A (orange arrow). A characteristic band at 3122 nm was observed in CNTs that were treated with HNO_3_ as illustrated in Figure S2A (black arrow). This characteristic band was due to the presence of the aromatic C–H group in CNTs treated with HNO_3_. 

The following characteristic band were observed in both SOCl_2_ and HNO_3_ treated CNTs; 3543, 3470, 3414 and 3236 cm^−1^. These characteristic bands were generated as a result of the O–H group from the water during CNTs solution preparation. The characteristic band at 2924 and 2850 nm were due to the C-H functional groups during a chemical reaction between CNTs and SOCl_2_ and HNO_3_. The characteristic bands that were observed at 1619, 1385, 1261, 1097 cm^−1^ were due to the C=C, C–H, C–C and C–O functional groups, respectively. In addition, the characteristic bands that were observed at 615 and 476 cm^−1^ were due to C–Cl groups (Figure S2A). These findings confirm the successful functionalisation of the MWCNTs with HNO_3_ and SOCl_2_.

#### 3.1.5. Raman Evaluation of the Functionalised MWCNTs

Raman analysis was performed in the functionalised MWCNTs using Raman Micro200. The D and G bands were observed at 1362 and 1568 nm, respectively. As depicted in Figure S2B, the G (graphene or graphite-like) band intensity is higher than the D (defect or disorder-induced) band intensity implying that the multiwalled carbon nanotubes were pure and even purer following purification and functionalization with HCl, SOCl_2_ and H_2_SO_4_. MWCNTSs functionalised with H_2_SO_4_ had the highest intensity compared to the rest. In addition, the H_2_SO_4_ treated MWCNTs had approximately the same G and D band intensities. This is indicative that the sulphuric acid functionalisation may have caused a defect in the structure of the CNTs as illustrated by an increase in the D band intensity. The intensity of the SOCl_2_ functionalised MWCNTs was greater than that of the pristine and HCl treated MWCNTs (Figure S2B). 

Raman spectra of FITC-DEX-PEG-ANP-CNTs were determined to confirm the conjugation of ANP antibody to the functionalized MWCNTs. The pristine MWCNTs had two bands namely D and G. The D (defect or disorder-induced) band and G (graphene or graphite-like) band at 1342 and 1580 cm^−1^, respectively, but their intensities were very low as compared to the functionalised CNTs. The D band is activated by disorders in the carbon systems. The G band with a shoulder around 1604 cm^−1^ is due to the in-plane vibration of the C–C bond in the CNTs. In this study, the D and G bands in the native MWCNTs were observed at 1300 and 1578 cm^−1^, respectively. The intensity for both the D and the G bands for the pristine MWCNTs was 1.87 × 10^3^ and 1.63 × 10^3^, respectively (Figure S2B). 

Dong and colleagues observed an increase in the intensity of the G band and no change in the intensity of the D band when a small amount of water was added to the reaction during floating catalyst CVD. The intensity increased when the amount of water added to the catalyst was increased. This CNTs quality improvement could be contributed by the effective catalyst cleaning by amorphous carbon removal [28]. 

**Figure 5:**
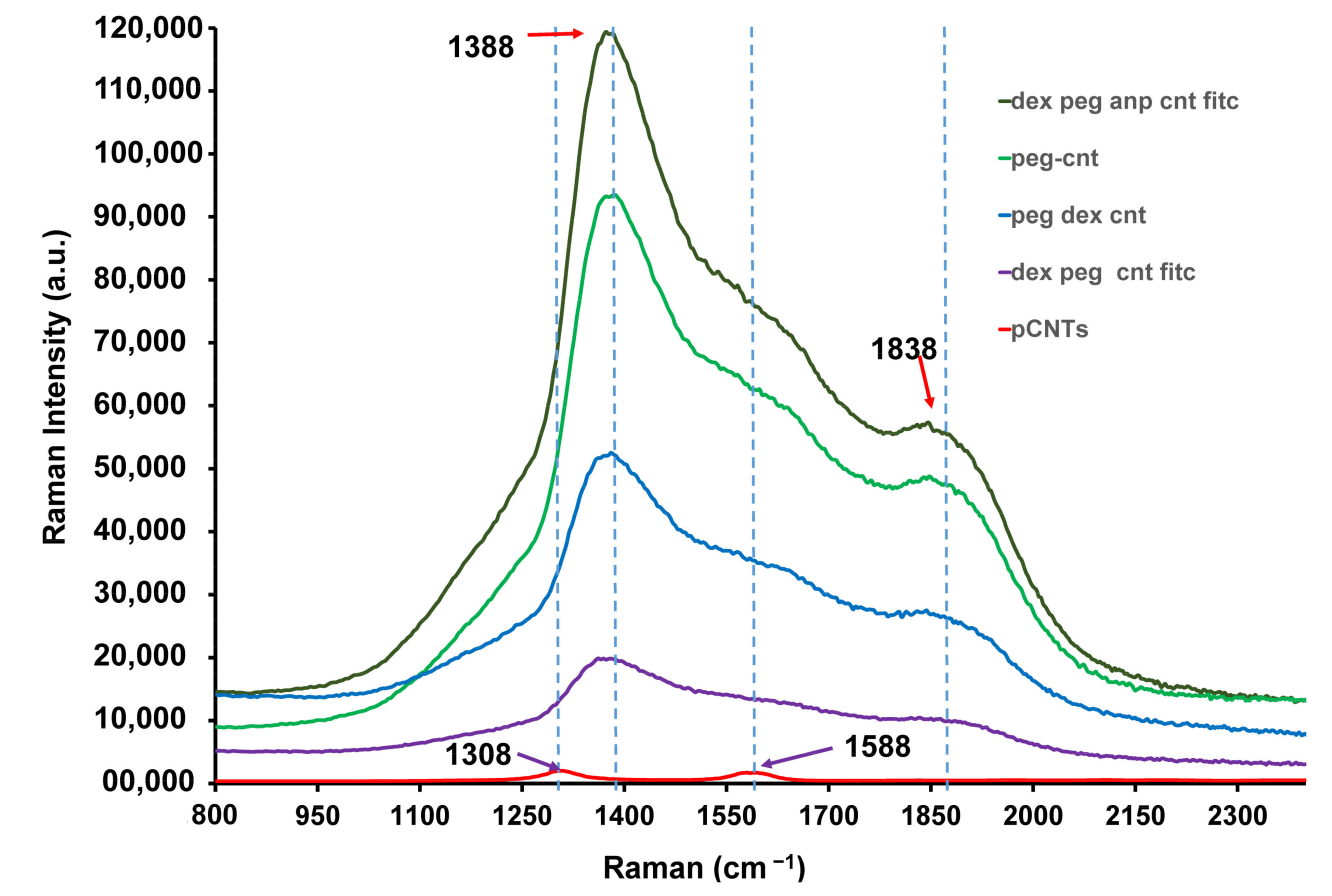
Raman spectra of the functionalised multiwalled carbon nanotubes. Raman spectra of FITC-DEX-PEG-ANP-CNTs, PEG-CNTs, DEX-PEG-CNTs, FITC-DEX-PEG-CNTs and pCNTs.

Following functionalisation with various molecules, both bands shifted to the right as depicted in Figure 5. Furthermore, the bands were widened and the resolution between these two bands was significantly affected. The D band intensity increased substantially compared to the G band intensity (Figure 5, Table 1).

Functionalisation increased the D band intensity of the pristine CNTs in FITC-DEX-PEG-CNTs, DEX PEG-CNTs, PEG-CNTs and FITC-DEX-PEG-ANP-CNTs by 10×, 27×, 49× and 63×, respectively. Moreover, the G band intensity of the pristine band in FITC-DEX-PEG-CNTs, DEX-PEG-CNTs, PEG-CNTs and FITC-DEX-PEG-ANP-CNTs were increased by 6×, 16×, 29× and 34×, respectively as illustrated in Table 1. These changes could be due to the covalent and non-covalent bonds that were generated during the chemical reactions taking place between the CNTs and the added molecules. The broadening and change in intensity confirmed the interaction between MWCNTs and new functional groups added to the CNTs. 

#### 3.1.6. UV–Vis Scanning and FEEM Evaluation of the FITC-DEX-PEG-ANP-CNTs

**Figure 6:**
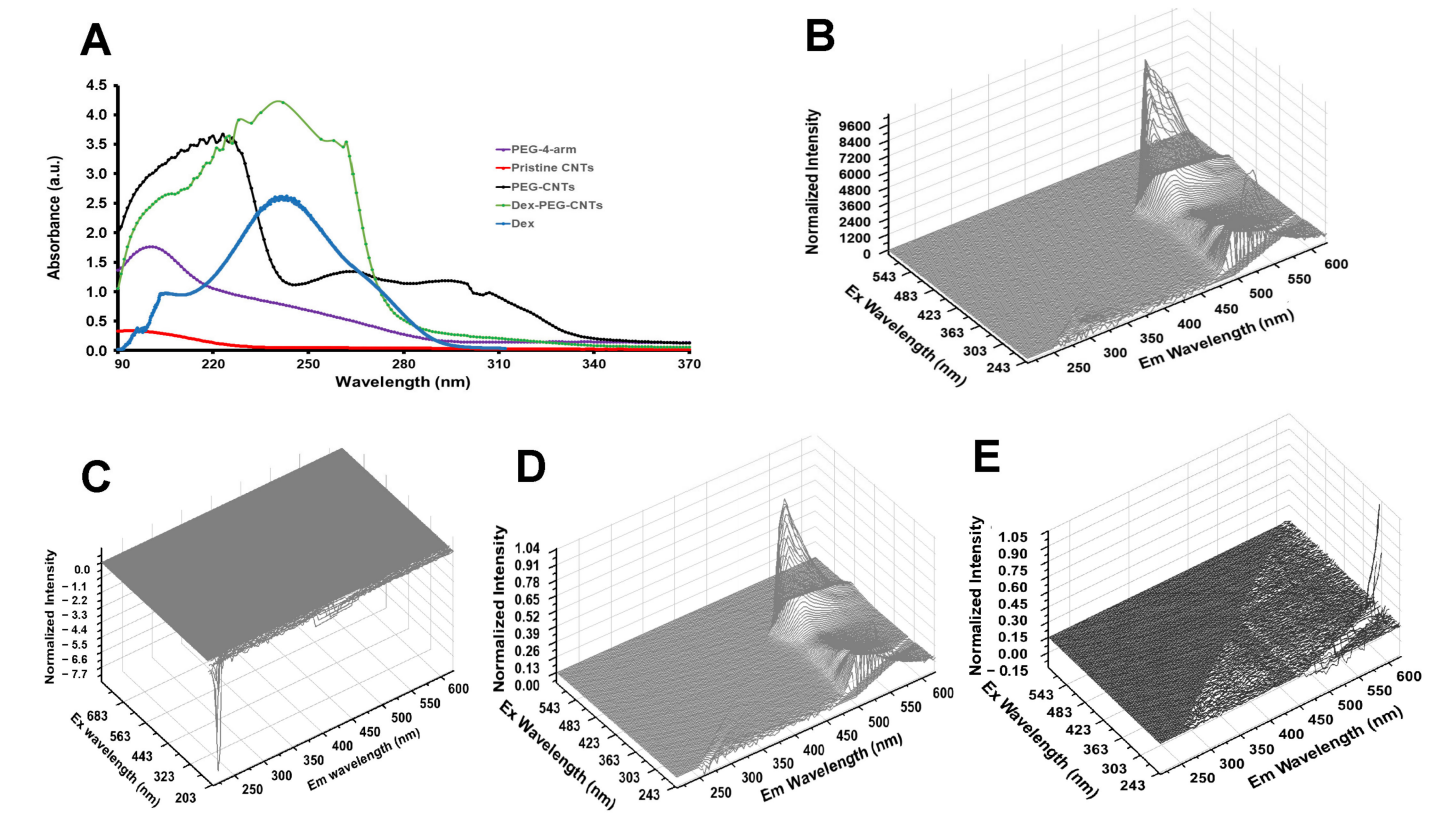
UV–Vis and FEEM spectra of the functionalised MWCNTs. UV–Vis spectra of PEG-4-arm, pristine pCNTs, PEG-CNTs, DEX-PEG-CNTs and DEX (**A**). FEEM spectra of FITC (**B**) and DEX-PEG-CNTs (**C**). FEEM spectra of FITC-DEX-PEG-CNTs (**D**) and FITC-DEX-PEG-ANP-CNTs (**E**).

Pristine carbon nanotubes are hydrophobic, and they were non-soluble in water as a result. Functionalisation degree and the type of solvent have a greater impact on their dispersibility in an aqueous milieu. HCl-treated CNTs were slightly soluble in water whereas H_2_SO_4_:HNO_3_ and SOCl_2_ functionalised carbon nanotubes were readily soluble in water. Functionalised CNTs were PEGylated and dexamethasone loaded on them. PEGylation is a covalent addition of non-toxic, non-immunogenic, and readily water-soluble PEG (Polyethylene glycol) to the molecule such as nanoparticles. This procedure was performed to reduce the degradation of the formulations via metabolic enzymes leading to the enhancement of the therapeutic potential of the complex by increasing its circulation time. They were resuspended in deionised water and sonicated for 5 min prior to UV–Vis analysis. The optimal wavelength of pristine MWCNTs was observed at 194 nm. In the PEG-CNTs and DEX-PEG-CNTs, the optical wavelengths were 225 nm and 245 nm, respectively. Additional characteristic bands at 265 and 295 nm were observed in PEG-CNTs and these could be attributed to the bonds formed by functional groups from PEGylation (Figure 6A). The optical wavelength relocated to the right of the native CNTs but within the UV range following functionalisation of CNTs with PEG and DEX as depicted in Figure 6A.

PEG-4-arm and DEX optical wavelength were observed at 201 nm and 242 nm, respectively. These results agree with the data of Roozbahani and colleagues. They encapsulated DEX into laponite nanoplatelets and performed UV–Vis scanning on DEX, DEX encapsulated nanoplatelets, and DEX free nanoplatelets. They observed a characteristic band at 242 nm in DEX and DEX encapsulated nanoplatelets. This characteristic band was not observed in DEX-free nanoplatelets. This is indicative that DEX was successfully encapsulated in their nanoplatelets [29]. 

In this study, the characteristic band of DEX around 242 nm was observed in DEX standard and DEX-PEG-CNTs. This characteristic band was not observed in pristine CNTs and DEX free PEG-CNTs. Functionalization altered the optical properties of the carbon nanotubes. A rise in absorbance could be attributed to the carboxylic and acyl groups that are added to MWCNTs and the size of the particles. These findings have illustrated the impact of functionalization of the carbon nanotubes on their optical characteristics as depicted in Figure 6A. 

The functionalised MWCNTs were scanned using Aqualog Horiba FEEM. The maximum excitation wavelength for FITC was observed at 490 nm and emission at 550 nm as depicted in Figure 6B. Fluorescence was not detected in DEX-PEG-CNTs as this complex was not labelled with a fluorescent tag (Figure 6C). Maximum excitation and emission wavelengths in FITC-DEX-PEG- CNTs complex were observed at 490 nm and 550 nm, respectively (Figure 6D). 

When ANP antibody was coupled to FITC-DEX-PEG-CNTs, excitation and emission were observed at 303 nm and 550 nm, respectively, but with very low intensity (Figure 3E). The interaction of ANP antibody with the FITC-PEG-CNTs seemed to have affected the fluorescence of the fluorescent tag. The fluorescence intensity was very low in this complex. Moreover, contour maps were also used to confirm the loading of the fluorescent tag to the carbon nanotubes as illustrated in Figure S3. FITC excitation wavelength and emission wavelength were observed at 490 nm and 520 nm, respectively (Figure S3A). DEX-PEG-CNTs did not express any fluorescence since there was no FITC labelling in this complex (Figure S3B). In FITC-DEX-PEG-CNTs, the excitation was at 490 nm and emission at 520 nm as a result of FITC that was attached to DEX-PEG-CNTs (Figure S3C). 

The contour map of the FITC-DEX-PEG-ANP-CNTs did not give a clear indication of the fluorescence. The formation of bonds between the ANP antibody and FITC-PEG-CNTs could have an impact on the FITC and affected its fluorescence to a certain extent as a result (Figure S3D). These findings confirmed the successful loading of FITC to DEX-PEG-ANP-CNTs. 

#### 3.1.7. Dexamethasone Loading and Release Studies

**Figure 7:**
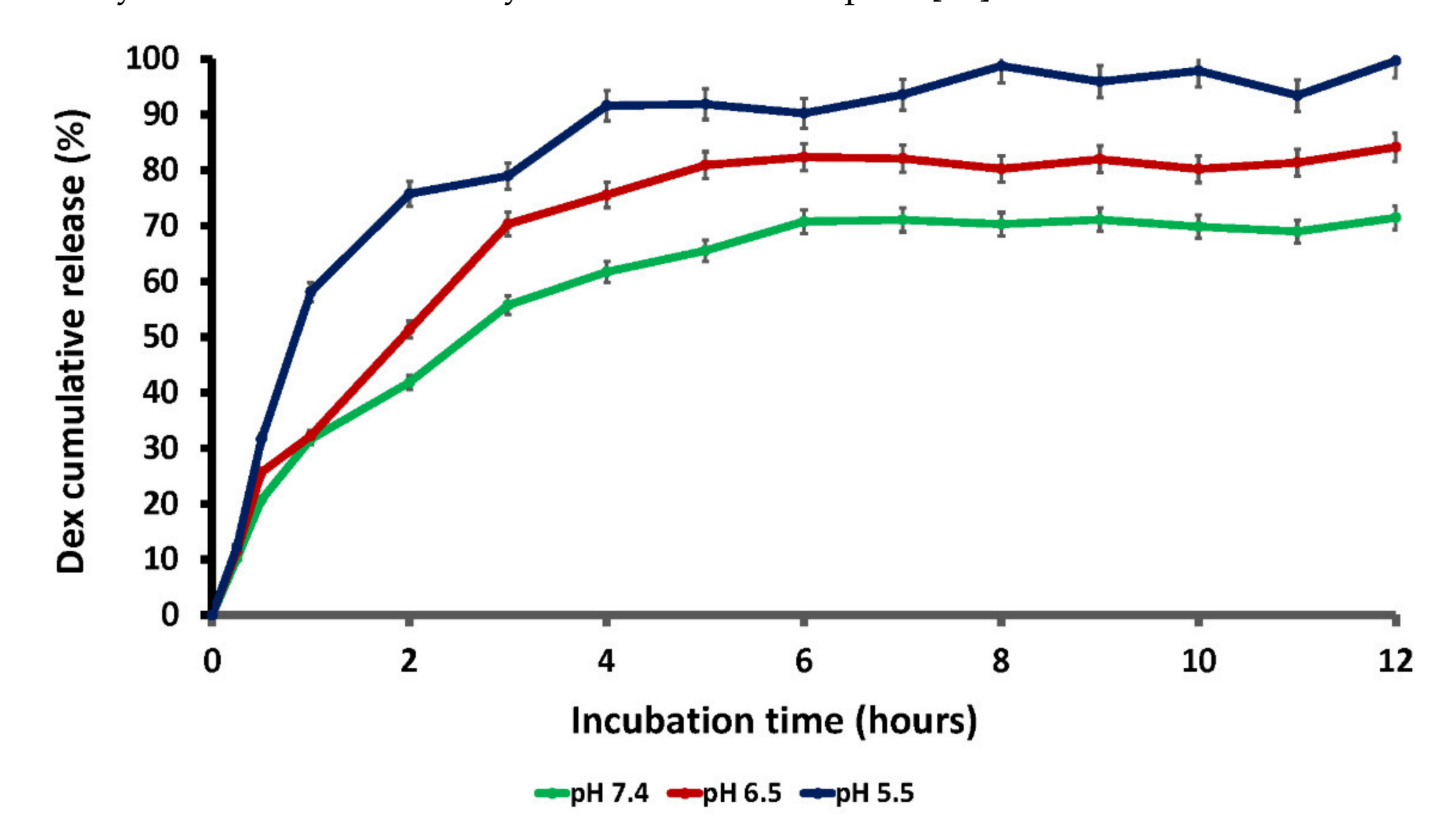
Dexamethasone release from the carbon nanotubes.

Dialysis tubing over 12 h at various time intervals indicated the initial fast release of dexamethasone from MWCNTs after 0.5 h. Maximum dexamethasone release occurred after 4 h and remained constant up to 12 h (Figure 7). The fast release of dexamethasone was observed after 2 h and the dexamethasone was gradually released until it reached a plateau after 4 h. This is indicative that dexamethasone was trapped inside the central cavity of the CNTs and slowly released as time elapsed [24].

There are two possible mechanisms that could have taken place: namely drug loading as a result of entrapment into the inner cavities of the carbon nanotubes and ionic interaction as a result of phosphate derivative. The mechanisms of dexamethasone release from PEGylated MWCNTs in an acidic and basic milieu have been demonstrated to be different. The burst and subsequent sustained release could play a vital role in the therapeutic treatments, as the initial fast release could rapidly afford a therapeutic dose, and the succeeding sustained-release could preserve the therapeutic dose for a prolonged period [23]. The above findings have demonstrated an improved sustained release of dexamethasone when loaded to the PEGylated MWCNTs.

#### 3.1.8. Fluorescence Evaluation of FITC-DEX-PEG-ANP-CNTs

The functionalised CNTs were labelled with FITC for easy tracking in different organs of the animal following intravenous injection. FITC is attached directly to the amino groups of the carbon nanotubes or amino groups of the DSPE-PEG5000-4-arm-(PEG-Amine) attached to CNTs or both. In this study, FITC excitation wavelength and emission wavelength were observed at 415 nm and 520 nm, respectively using the LS45 Fluorescence Spectrometer (PerkinElmer, Beaconsfield, UK) as depicted in Figure S3E. DEX-PEG-CNTs was fluorescently labelled with FITC and the excitation wavelength and emission wavelength were 263 nm and 500 nm, respectively (Figure S3F). 

When ANP antibody was coupled to FITC-DEX-PEG-CNTs, excitation and emission wavelength were shifted to the right to 330 nm and 660 nm, respectively, as a result of an interaction of ANP antibody with the complex (Figure S3G). These findings have demonstrated a successful loading of FITC to DEX-PEG- CNTs and DEX-PEG-ANP-CNTs.

### 3.2. Biological Properties of the Carbon Nanotube System

#### 3.2.1. Cytotoxic Effect of the FITC-PEG-ANP-CNTs in PC-12 Cells

**Figure 8:**
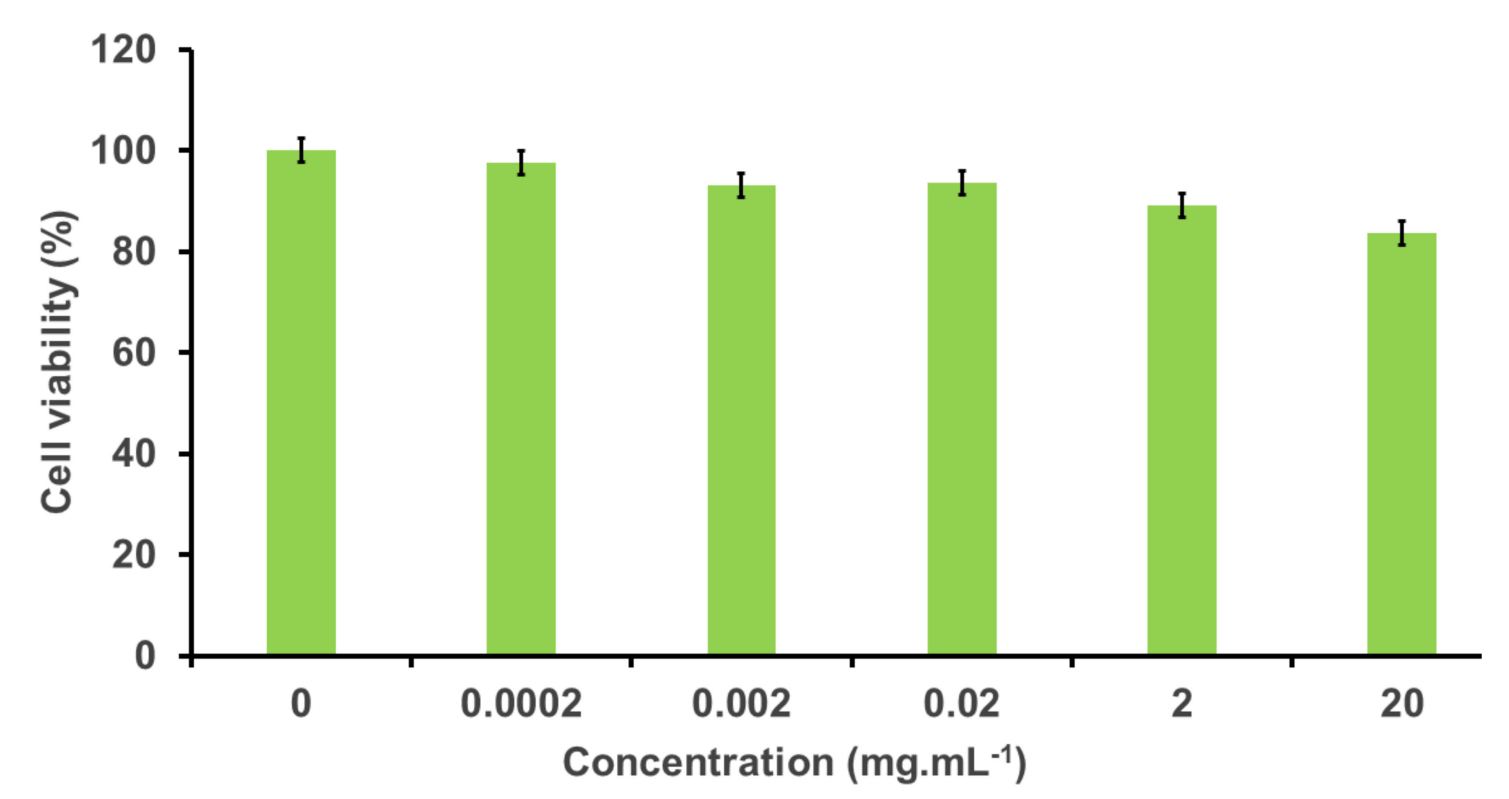
Cytotoxic effect of FITC-DEX-PEG-ANP-CNTs in PC-12 cells.

Cytotoxicity studies were performed to confirm the cytotoxic effect of the FITC-Dex-PEG. Different concentrations of FITC-DEX-PEG-ANP-CNTs were used and the viability was 80–100%. Our findings have demonstrated the reduction of cell viability with an increase in the concentration of the formulation as demonstrated by a slight decrease in the viability when a concentration range of 0–20 µg/mL of the formulation was used (Figure 8).

Yuan and colleagues evaluated the effects of MWCNTs on macrophages and found that long MWCNTs were more toxic than short MWCNTs [29]. As these carbon nanotubes were suspended in culture medium and ultrasonicated prior to use, the toxicity could be due to the fact that CNTs were not functionalized in Yuan’s study.

#### 3.2.2. Animal Surgery and Neurobehavioural Assessment

Occlusion of the common carotid artery to induce stroke was successfully performed and no mortality was reported. Tail venous blood was collected before and after surgery for the determination of the atrial natriuretic peptide. Formulations were administered intravenously on the tail immediately after blood was collected from the tail following surgery. An improved and swift recovery was observed in the group treated with FITC-DEX-PEG-ANP-CNTs compared to the other groups. 

**Figure 9:**
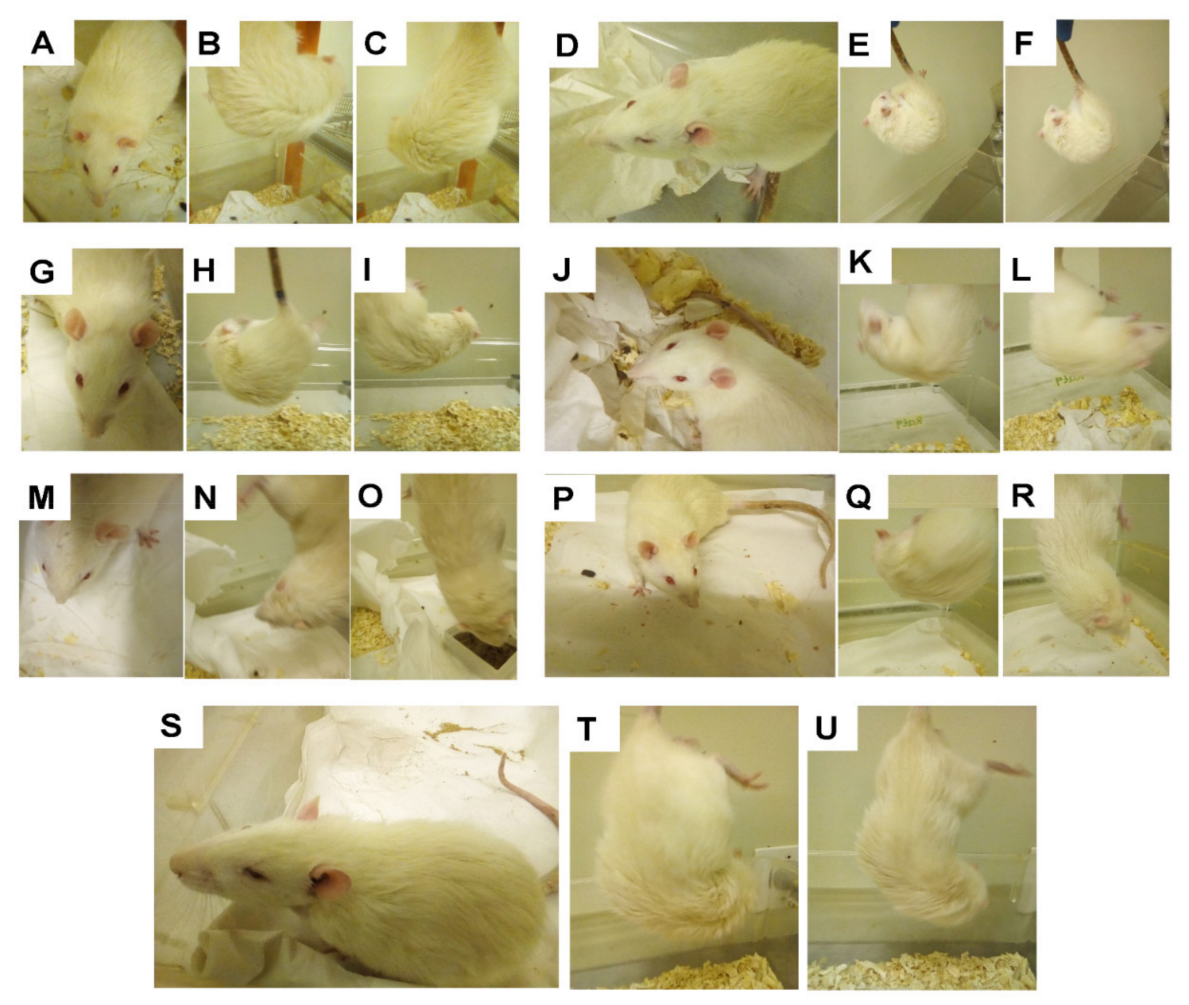
Neurobehavioural assessment of the Sprague Dawley rats following treatment with the formulations. Premedication rats were perfectly healthy with intact eyes (**A**), left hand side circling (**B**) and right hand side circling (**C**). CCAO rats with left eye sunken and dark red (**D**), right hand side circling only (**E**,**F**). FITC-DEX-PEG-ANP-CNTs treated group with improved eye condition, not sunken and slightly red (**G**). Both right and left hand side circling (**H**,**I**). Healthy non-ischemic and untreated rats with healthy intact eyes (**J**). Circling in both directions (**K**,**L**). PEG-CNTs treated rats with healthy intact eyes (**M**) and left and right hand side circling (**N**,**O**). Sham group with perfect and healthy eyes (**P**) and circling in both directions (**Q**,**R**). CCAO rat treated with DEX and the right eye sunken and dark red (**S**) and left hand side circling only (**T**,**U**).

It could therefore be deduced that FITC-DEX-PEG-ANP-CNTs played a pivotal role in the reduction of the symptoms of ischemic stroke as compared to the PEG-CNTs group as depicted in Table 2. There was no improvement in the health of the rats when dexamethasone alone was used. In addition, there was a speedy recovery from anaesthesia and higher BAR (Bright, Alert and Responsive) in the FITC-DEX-PEG-ANP-CNTs treated group as opposed to the other groups. Surgery was not conducted in the premedication group. The rats were injected with fentanyl, dormicum, temgesic, and lignocaine, and anaesthesia was maintained under isoflurane and oxygen. Both eyes were found to be intact and healthy. The fur was smooth, and ears raised up. The whiskers were fine and they were fully alert with a perfect BAR (Figure 9A, Table 2). The rats were circling to both sides and in all directions (Figure 9B,C). It could be deduced from the results obtained that premedication did not pose any observable detrimental effect on the rats. The left eye was sunken and it turned dark red (Figure 9D). 

Contralateral circling to the right when the rats were suspended by the tail was noticed since the left common carotid artery was occluded (Figure 9E,F and Table 2). The back arch and hunched posture were noticed. These symptoms are indicative of the stroke that was induced by occlusion of the common carotid artery. There was an improvement in the eyesight as both eyes were healthy. The left eye was not sunken but was dark red and returned to normal after 5 h of reperfusion (Figure 9G). The rat circled both sides and in all directions when it was suspended by the tail (Figure 9H,I and Table 2). 

This is indicative of the effect of FITC-DEX-PEG-ANP-CNTs on the health status of the rat. This implies that FITC-DEX-PEG-ANP-CNTs treated the stroke successfully in this group. Gholamine and colleagues evaluated the neurobehavioural toxicity of single-walled (SWCNTs) and multiwalled carbon nanotubes (MWCNTs) in mice. They found that CNTs could cause behavioural toxicity such as anxiety and depression depending on their structure. Furthermore, they found that MWCNTs were more toxic than SWCNTs [30]. In their study, carbon nanotubes were not functionalised and were also suspended in PBS and sonicated prior to use. 

In this study, cytotoxicity was not observed as the CNTs were functionalised and PEGylated prior to use (Figure 8). There is currently a debate on the toxicity of carbon nanotubes in animals hindering their clinical application [16]. Functionalised carbon nanotubes are apparently less toxic compared to pristine carbon nanotubes. In this study, pristine CNTs were not used as they were floating on top of the PBS. The functionalised CNTs were, however, well dispersed in the buffer. The functionalised CNTs were PEGylated to enhance their efficient delivery to the ischemic site as the PEGylation avoids opsonisation by macrophages.

PEG is responsible for enhancing the therapeutic potential of drugs by increasing its circulation time, decreases the immunogenicity, antigenicity of drugs, and is also responsible for the stability of drugs [31]. This group of rats was assessed prior to surgery during the habituation process. The fur was smooth, and both eyes were bright and reddish-pink (Figure 9J). The rats were fully alert and spontaneous movement in all directions was observed. The rats were circling in both directions (Figure 9K,L and Table 2). In general, the rats were healthy as there were no signs of stress nor symptoms of a stroke. There was an improvement in the general health of the rat. Both eyes were healthy and the rats were alert, and the fur was smooth. Spontaneous movement in all directions and spontaneous circling in both directions was noticed (Figure 9M–O and Table 2). The improvement in the healthy state of this group is indicative that the PEGylated CNTs could reduce inflammation in ischemic stroke leading to a healthy state of the rat. This also implies that PEG facilitated the delivery of the CNTs to the ischemic site [32]. From the results obtained, it could be deduced that CNTs have a beneficial role in the healing of stroke. 

In the sham group, the common carotid artery was isolated but not occluded. Following isolation of the common carotid artery, the wound was closed after 30 min. Since the artery was not occluded, the blood supply to the brain was not interrupted. The rats were healthy with bright and reddish-pink eyes (Figure 9P). The fur was smooth, and no back arch or hunched posture was noticed in this group. Circling was spontaneous on both sides and spontaneous movement in all directions was noticed (Figure 9Q,R). 

In the DEX treated group, the right eye was dark red and sunken. The rat had a back arch and hunch posture and did not look healthy (Figure 9S). Spontaneous movement in all directions and spontaneous circling to both directions were not observed. The rat circled to the right-hand side only (Figure 9T,U). From the results observed, it seems that DEX alone did not have any beneficial impact on the health of the rat (Table 2).

#### 3.2.3. Humane Endpoints and Sampling

The rats underwent 24-h reperfusion following surgery and i.v. injection with the formulations. Reperfusion injury was observed in stroke-induced rats that were not offered any treatment [33]. Venous blood withdrawn from the tail (before surgery and after surgery) and blood from the heart (after 24-h reperfusion) were centrifuged to separate plasma from the red blood cells (Figure S4A,B). Plasma was successfully separated from the red blood cells and stored at −20 °C for atrial natriuretic peptide measurements. Rats were euthanized following 24-h reperfusion, cardiac puncture was performed, and phosphate-buffered saline perfusion was also carried out [34]. A cardiac puncture was performed to obtain a large volume of blood for a variety of measurement techniques. The brain was successfully isolated from the skull for the weight measurements. The olfactory bulbs and cerebellum were removed from the brain and the volume of the brain was determined and was approximately 2 mL (Figure S4C).

Brain hemispheres were successfully separated, weighed, and stored in 10% formalin to prevent putrefaction and to maintain the anatomical architecture of the organs. The mechanism of action of fixation is to terminate enzymatic reactions and metabolic activities by denaturing intrinsic biomolecules. Proteolytic enzymes that digest the tissue via autolysis are denatured, and autolytic processes are terminated. Fixatives protect the tissues from extrinsic damage as they are toxic to most microorganisms. Furthermore, many fixatives chemically alter the treated tissue to be less palatable to microorganisms, thereby hindering putrefaction [35]. Perfusion was successful in all organs as depicted by a colour change from red to pale following perfusion with phosphate-buffered saline (Figure S4C). The other organs such as the spleen, heart, liver, kidneys, and lungs were properly perfused and successfully isolated as demonstrated in Figure S5A. The spleen was still red as it is the blood filter and old red blood cells are recycled in this vital organ and it was not easy to perfuse it completely. The harvested organs were stored in 10% formalin to prevent putrefaction to preserve their anatomical morphology for histological evaluation depicted in Figure S5A [36].

#### 3.2.4. Brain and Total Body Weights following Common Carotid Artery Occlusion and Treatment with the Formulations

Fabian and colleagues studied the effect of PEGylated hydrophilic carbon clusters (PEG-HCC) in hyperglycemic ischemic stroke-induced Sprague-Dawley rats. These nanoparticles led to a reduction in infarct volume and hemisphere swelling [37]. In this study, functionalised MWCNTs were used to study their effects in the treatment of stroke in different treatment groups.

**Figure 10:**
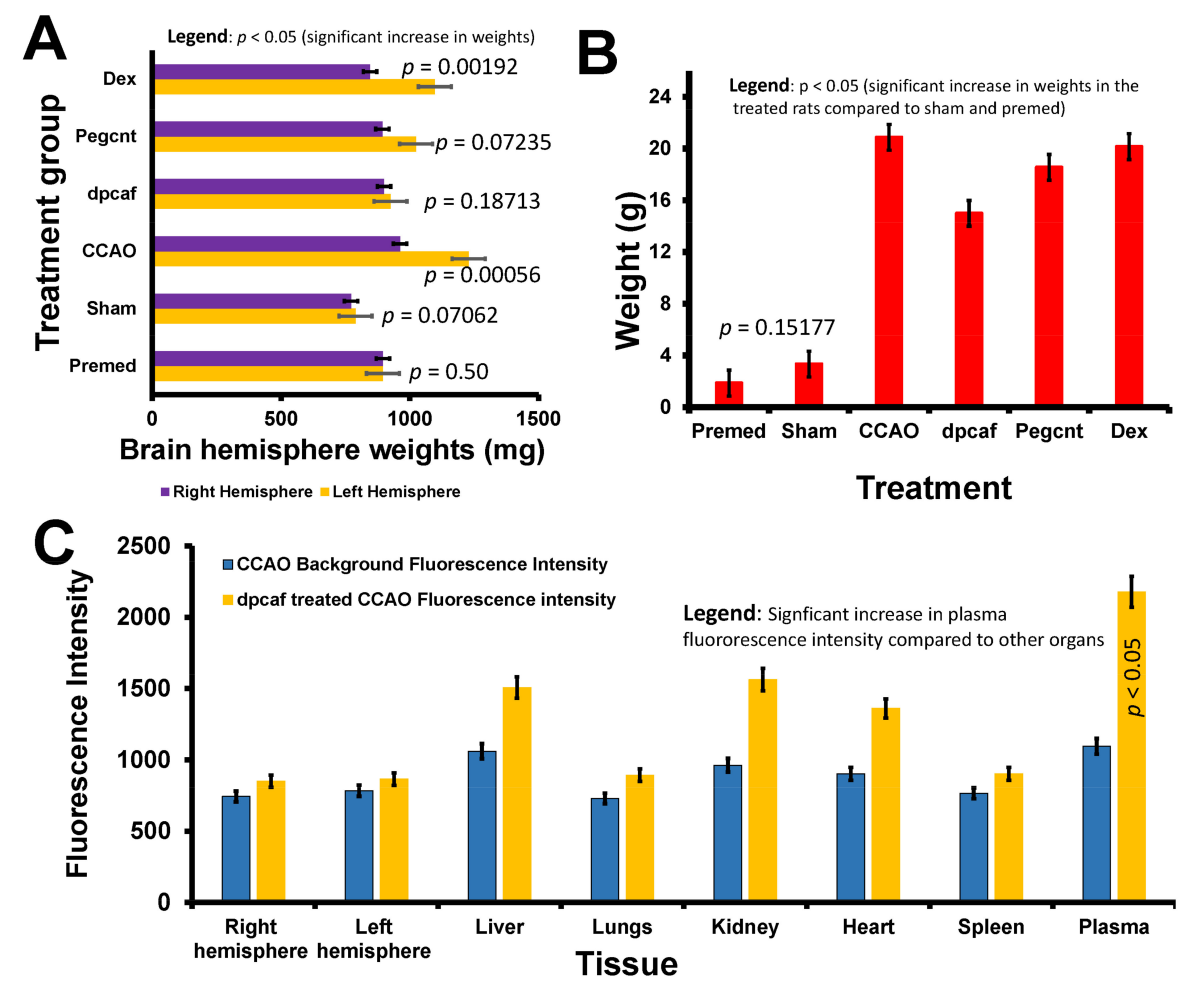
Total body and brain hemisphere weights following treatment of rats with the formulations. Brain hemisphere weights before and after treatment of the rat with the formulations (**A**). Total body weight loss during CCAO and following treatment (**B**). Average fluorescence intensities in various organs in non-treated and FITC-DEX-PEG-ANP-CNTs treated CCAO rats (**C**).

In the Premed group, there was no change in both the left and the right brain hemisphere weights as the rat did not undergo any surgery (*p* = 0.50) as illustrated in Figure 10A. In the sham group, there was a very slight difference in the hemisphere weights (*p* = 0.07062) and this could be due to the stress as surgery was performed but there was no occlusion of the vessel (Figure 10A). A vast difference in hemisphere weights was observed in the common carotid artery occlusion group. 

Since the occlusion was performed in the left common carotid artery, the rise in weight of the left hemisphere was highly noticeable. The weight increased as a result of inflammation that was caused by an induction of stroke. This is indicative of the brain oedema that was generated during stroke induction. From the graph, it could be deduced that brain oedema was triggered by common carotid artery occlusion as demonstrated by the increase in the weight of the left hemispheres confirming the presence of ischemic stroke in the rats. 

The left hemisphere weight in the rats that underwent common carotid artery occlusion increased significantly (*p* = 0.00056) due to the inflammation that was triggered by artery occlusion. The weight of this hemisphere was reduced significantly to almost the weight of the right hemisphere (*p* = 0.18713) following treatment with FITC-DEX-PEG-ANP-CNTs. This implies that the FITC-DEX-PEG-ANP-CNTs formulation reduced the inflammation in the left hemisphere as demonstrated by a drastic drop in the weight of the right hemisphere in the FITC-DEX-PEG-ANP-CNTs group (Figure 10A). The left hemisphere weights of the CCAO rats were slightly reduced in the PEG-CNTs group (*p* = 0.07235). The data has demonstrated that PEG-CNTs reduced the inflammation in the CCAO rat but not effective as compared to the FITC-DEX-PEG-ANP-CNTs (Figure 10A). 

The reduction in the inflammation could be due to the anti-inflammatory properties of the CNTs that reached the ischemic site. The PEG facilitated the smooth delivery of the CNTs to the site. There was no reduction in the inflammation in the left hemisphere in the rats that were treated with DEX only as confirmed by a significant difference (*p* = 0.00192) in the weights following treatment with DEX (Figure 10A). Although DEX is well-known for its anti-inflammatory properties, it could not reduce the inflammation, and this could be due to its low dose affecting its availability at the ischemic site. There was no significant decrease in the weights of the left hemispheres in both the sham (*p* = 0.07062) and premed (*p* = 0.50) groups.

In order to check whether the rats were losing weight as a result of the intervention, their pre-surgery and post-reperfusion total body weights were recorded. The weight loss of the premed group was very minimal as compared to the other groups. In the sham group, there was a slight increase in the weight loss and this could be due to the stress during surgery to expose the common carotid artery. There was no significant difference in weight loss when sham and premed groups were compared (*p* = 0.15177) as demonstrated in Figure 10B. There was a drastic increase in the weight loss in the rats following the common carotid artery occlusion as confirmed by a comparison of the premed and the common carotid artery occlusion group (Figure 10B).

The rats that underwent common carotid artery occlusion experienced a lot of stress and this led to the increase in weight loss (*p* < 0.05). Furthermore, mastication and swallowing functions were negatively impacted and these could have led to restricted food ingestion and water intake and resulted in postsurgical body weight loss and aggravated motor performance [38]. A drop in weight loss was observed following treatment of the rat with FITC-DEX-PEG-ANP-CNTs. A small drop in loss of weight was noticed following the treatment of the rat with PEG-CNTs. There was a small drop of mass in the DEX treated rats (Figure 10B). These results have demonstrated the beneficial effect of both the FITC-DEX-PEG-ANP-CNTs and PEG-CNTs in the monitoring of weight loss during induction of stroke.

#### 3.2.5. Biodistribution of FITC-DEX-PEG-ANP-CNTs Using Absorption Spectroscopy

Various concentrations of FITC-DEX-PEG-ANP-CNTs ranging from 0–100 µg/mL were prepared and scanned at 490 nm using a Perkin Elmer multilabel reader Victor X3. A calibration curve was constructed and concentrations of FITC-DEX-PEG-ANP-CNTs of the samples from different tissues were determined by extrapolation of the standard curve (Figure S5B). The absorbance was measured from different tissues such as the liver, kidney, lungs, brain, blood, and spleen. 

The highest levels of FITC-DEX-PEG-ANP-CNTs were found in the spleen and liver with 49.66 and 29.42 µg/mL, respectively. This is indicative that the higher percentage of the formulation was absorbed by the spleen and liver as compared to the rest of the tissues due to their structural setting. 

The least concentrations of FITC-DEX-PEG-ANP-CNTs were recorded in the heart and lungs with 3.79 and 7.78 µg/mL, respectively. The levels of FITC-DEX-PEG-ANP-CNTs in the left hemisphere were higher than in the right hemisphere with 22.63 and 18.82 µg/mL, respectively. Stroke weakens the integrity of BBB and this could enhance the delivery of drugs and other biomolecules to the ischemic site [39]. The higher levels of FITC-DEX-PEG-ANP-CNTs in the left hemisphere could be due to the successful targeted delivery of FITC-DEX-PEG-ANP-CNTs to the ischemic site as the anatomy of the BBB was adversely impacted by stroke.

#### 3.2.6. Biodistribution of FITC-DEX-PEG-ANP-CNTs Using Fluorescence Spectroscopy

The native fluorescence, commonly known as auto-fluorescence is a widespread phenomenon and is caused by the presence of intrinsic biomolecules acting as endogenous fluorophores in living organisms. Brain tissue possesses autofluorescing molecules that interfere with neuronal cells identification when observed under fluorescent microscopy [40]. 

In this study, fluorescence was detected in the rat organs that were treated with FITC labelled formulations. Background fluorescence was also detected in rat organs that were not treated with FITC labelled formulations. This background fluorescence was caused by the auto-fluorescence from the animal tissues due to the presence of endogenous fluorophores. The background fluorescence was very minimal as black 96-well plates were used to reduce the fluorescence in this work. The fluorescence intensity calculations in all the tissues were determined using MyAssays software. The fluorescence was higher in the organs of the FITC-DEX-PEG-ANP-CNTs treated group since the FITC was incorporated in the formulation for tracking the location of the complex in various organs. The biodistribution of silica nanoparticles demonstrates their high accumulation in organs of the reticuloendothelial system such as liver and spleen [41]. In this study, the highest fluorescence was observed in plasma followed by kidney, liver, and heart (i.e., plasma > kidney > liver > heart). There was not much difference in the fluorescence intensity in the lungs, spleen and brain as illustrated in Figure 10C. 

The high level of fluorescence intensity in plasma compared to other organs is indicative that the drug was still in the blood and not completely cleared out of the blood circulation. This implies that a 24-h reperfusion period was not enough to clear the drug out of circulation. More time was needed for the complete delivery of the drug to the brain and clearance out of the circulation. It could be that it was slowly released, and this could extend its time in the circulation to improve bioavailability. The formulation was delivered to all the organs as demonstrated by the presence of fluorescence intensity in all the organs (Figure 10C). 

#### 3.2.7. Determination of Plasma ANP Levels in the Rat Venous Blood

Physiological ANP levels are very low in the non-ischemic healthy rat. The levels are elevated when the rat is under stress such as ischemic stroke. When the cardiac muscle is distended, ANP and BNP are released. ANP and BNP participate in the long-term monitoring of sodium and water balance, blood volume, and arterial pressure. These natriuretic peptides stimulate vasodilation and thereby decrease blood pressure. The NPs increases the glomerular filtration rate (GFR) leading to stimulation of natriuresis and diuresis [42]. Furthermore, NPs prohibit the release of renin leading to a drop in angiotensin II and aldosterone which induces further natriuresis and diuresis (Figure S5C).

**Figure 11:**
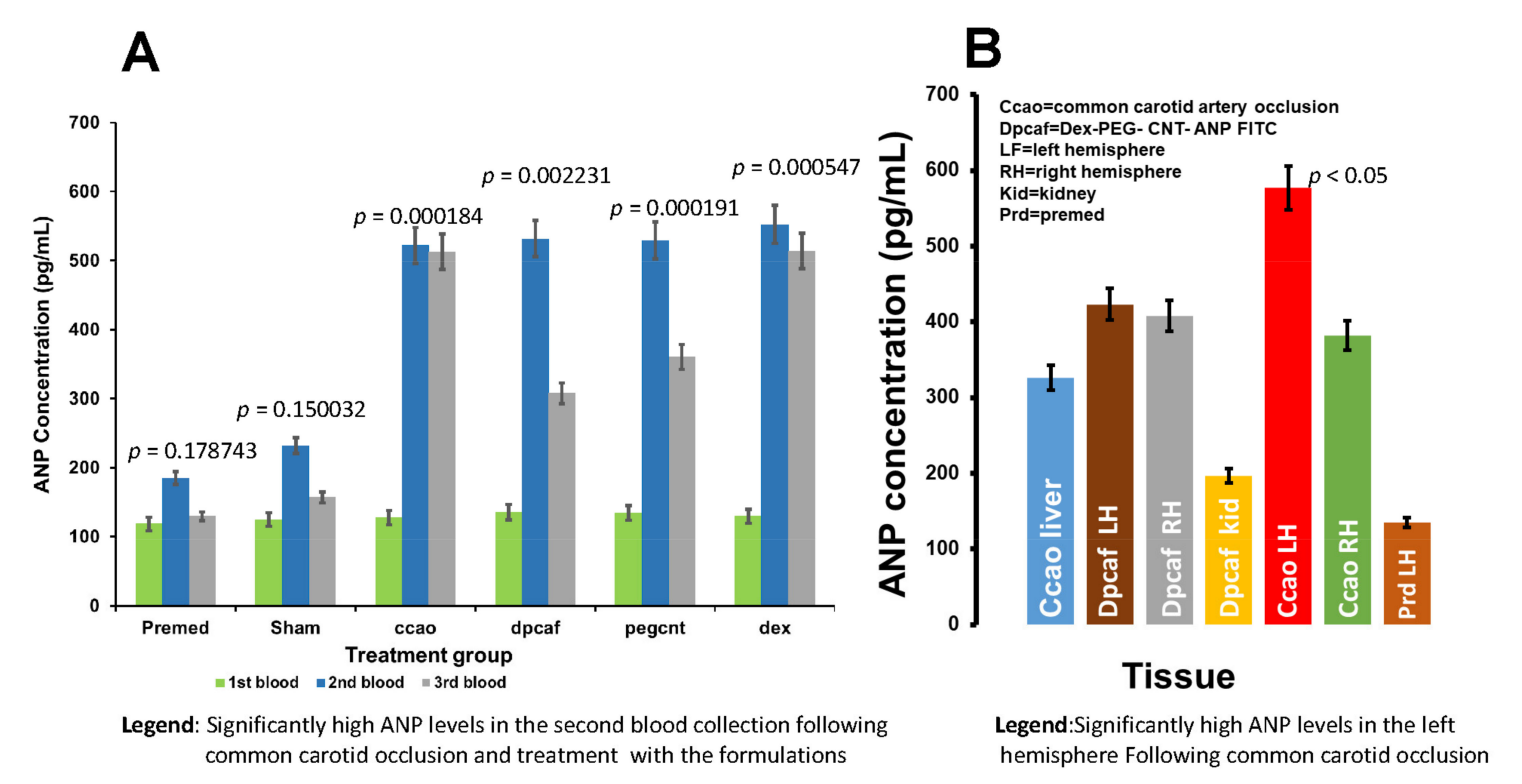
ANP concentrations in rat plasma and tissues. Rat Plasma ANP levels from first, second and third blood withdrawal before and after surgery including 24-reperfusion (**A**). ANP levels in different tissues of the treated and untreated rats (**B**).

Makikallio and colleagues measured the level of ANP in patients with stroke and reported the ANP levels as highly elevated [43]. In this study, plasma ANP levels were measured before CCAO, immediately after CCAO, and after 24 h of reperfusion (Figure 11A). Baseline levels of plasma ANP were around 100 pg/mL. ANP levels increased significantly following CCAO (*p* = 0.000184) and decreased significantly when rats were treated with FITC-DEX-PEG-ANP-CNTs (*p* = 0.002231) and PEG-CNTs (*p* = 0.000191) as illustrated in Figure 11A. An increase in ANP levels in the CCAO group was due to stroke that was induced in this group. 

Levels of ANP were very low in healthy rats but increased when the rats were under stress such as stroke to suppress inflammation. These data have demonstrated the effectiveness of PEG-CNTs and FITC-DEX-PEG-ANP-CNTs in reducing inflammation in stroke-induced rats as demonstrated by the significant drop in the levels of ANP in FITC-DEX-PEG-ANP-CNTs (*p* = 0.002231) and PEG-CNTs (*p* = 0.000191) treated rats. The levels of ANP depend on many factors such as tissue type, age, health status, and gender. In this study, male rats were used and ANP levels were determined in various organs. 

López-Morales and colleagues investigated the neuroprotective effect of ANP in rats. Three groups of rats were subjected to MCAO and received low-dose ANP and high-dose ANP following reperfusion. A neurobehavioural evaluation was performed and brain infarct and oedema volumes were measured following MCAO. A significant improvement in neurobehavioural condition was observed and the reduction in brain infarct and oedema volumes was demonstrated in the high-dose ANP group [44]. In this study, the levels were lower in the premed group as stroke was not induced in this group (Figure 10B) (tan bar). ANP was detected in the kidneys from the CCAO FITC-DEX-PEG-ANP-CNTs treated group but slightly higher than in the premed group (Figure 10B) (yellow bar). The highest levels of ANP were found in the CCAO group without treatment as a result of the stroke that was induced in the left hemisphere (Figure 10B) (red bar). ANP levels were elevated to reduce inflammation caused by stroke. 

Since the ANP has neuroprotective properties, ANP levels in the right hemisphere were lower than in the left hemisphere with stroke as it was not induced in the right hemisphere (Figure 11B) (green bar). A reduction in the ANP levels was observed in the FITC-DEX-PEG-ANP-CNTs treated groups (Figure 11B) (blue, brown and grey bars). This is indicative that the formulation was effective in reducing inflammation hence a drop in ANP levels.

#### 3.2.8. Histological Evaluation of FITC-DEX-PEG-ANP-CNTs Delivery into the Brain by Fluorescence Microscopy

A drug of interest (dexamethasone) is coupled to a carbon nanotube (multiwalled carbon nanotube). A targeting ligand (ANP antibody) is attached to the carbon nanotube for targeting the site of interest (ischemic site with a high concentration of ANP). A fluorescent tag is also coupled to the carbon nanotube either externally or internally for localization of the targeting ligand-drug complex. 

The complex is injected into the stroke-induced rat via the tail vein. The complex will circulate in the blood until it reaches the ischemic site where ANP is concentrated. The ANP antibody will bind to the ANP and dexamethasone will reduce inflammation on that site. The site can be located by the presence of FITC that was attached to the complex. Blood is then withdrawn and the amount of ANP that was synthesised before, after stroke induction and post-stroke treatment is determined by ELISA. 

Delivery of FITC-DEX-PEG-ANP-CNTs in the brain was further confirmed by fluorescence microscopy, Zeiss LSM 780 Confocal Microscope with Airyscan (Zeiss, Gottingen, Germany).

The fluorescence detected in the premedication group was due to the tissue fluorescence background (auto-fluorescence) but it was very low as black 96-well plates were used to reduce the background. Cells fluoresce when exposed to UV/Vis radiation of a specific wavelength due to their endogenous fluorophores [45]. Auto-fluorescence impedes accurate identification of cellular and subcellular components of the tissues. In this study, the intensity of fluorescence was very low as demonstrated by the darker background in the premed group compared to the treated groups. 

**Figure 12:**
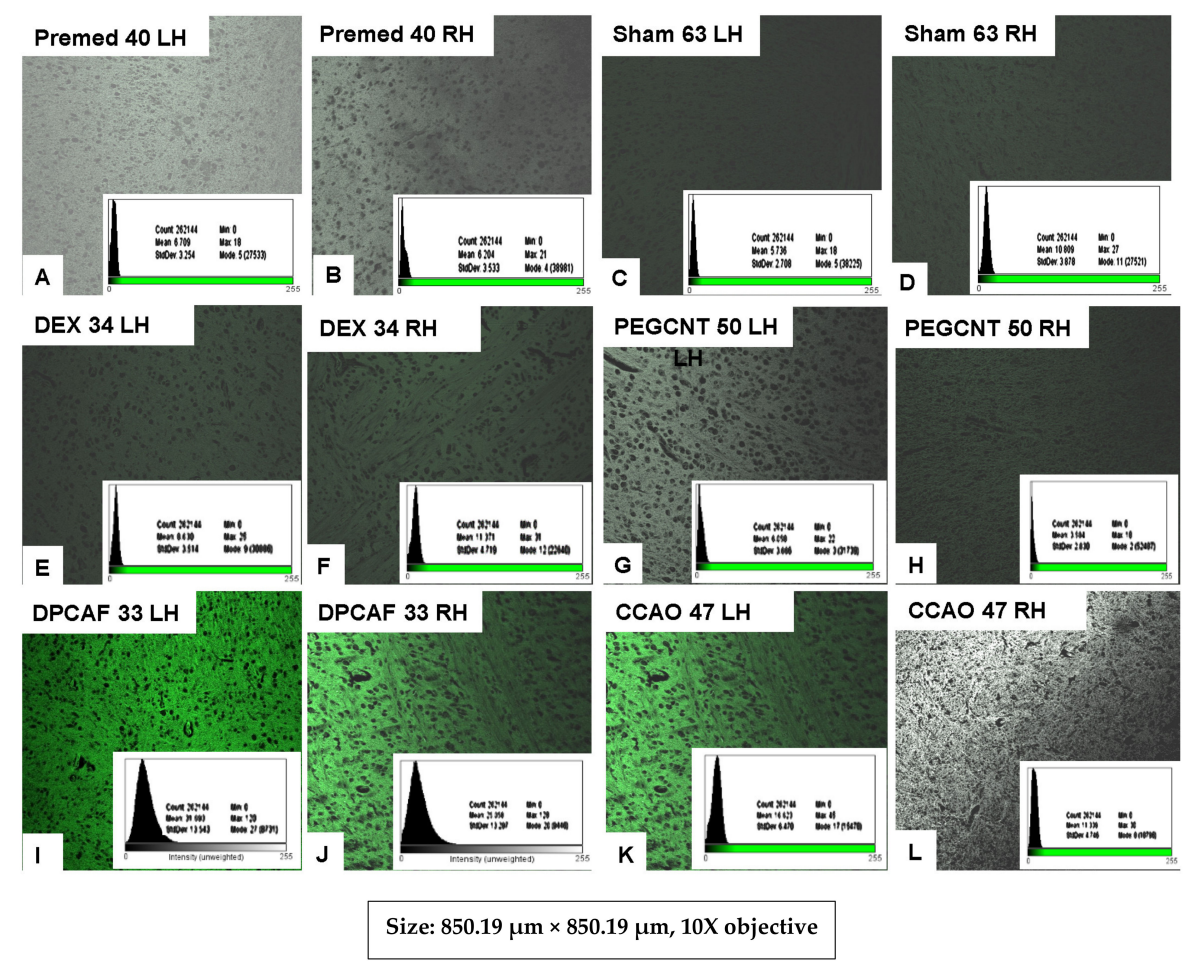
Fluorescence Intensities in the brain tissues of the treated Sprague Dawley Rats. Size (850.19 µm). (**A**). Premed 40 LH (Left hand side hemisphere of rat #40 treated with fentanyl, dormicum, temgesic and lignocaine). (**B**). Premed 40 RH (Right hand side hemisphere of rat #40 treated with fentanyl, dormicum, temgesic and lignocaine). (**C**). Sham 63 LH (Left hand side hemisphere of rat #63 with isolated common carotid artery but not occluded). (**D**). Sham 63 RH (Right hand side hemisphere of rat #63 with isolated common carotid artery but not occluded). (**E**). Dex 34 LH (Left hand side hemisphere of rat # 34 treated with DEX). (**F**). Dex 34 RH (Right hand side hemisphere of rat # 34 treated with DEX). (**G**). PEGCNT 50 LH (Left hand side hemisphere of rat # 50 treated with PEGCNT). (**H**). PEGCNT 50 RH (Right hand side hemisphere of rat # 50 treated with PEGCNT). (**I**). DPCAF 33 LH (Left hand side of rat # 33 treated with PEGCNT). (**J**). PEGCNT 33 RH (RH hand side hemisphere of rat # 33 treated with PEGCNT). (**K**). CCAO 47 LH (Left hand side of rat # 47 with common carotid artery isolated and occluded, no treatment). (**L**). CCAO 47 RH (Right hand side hemisphere of rat # 47 with common carotid artery isolated and occluded, no treatment).

The fluorescence intensity increased in the CCAO and FITC-DEX-PEG-ANP-CNTs treated groups. The fluorescence intensity in the left hemisphere was higher than that of the right hemisphere as illustrated in Figure 12. The difference in the intensity could be due to the fact that stroke was induced in the left hemisphere hence higher intensity of fluorescence. The complex had the anti-ANP antibody attached to it and was transported to the diseased site and bound to the ANP molecule as its level was increased in the site of ischemia. This is indicative of the successful delivery of the drug to the site of interest in the brain.

#### 3.2.9. Histological Evaluation of FITC-DEX-PEG-ANP-CNTs Delivery into the Brain by Hematoxylin and Eosin Staining Technique

Ischemic stroke is a disease of the cerebrovascular system and includes risks such as apoptosis, necrosis and cerebral infarction [46]. Yang and colleagues determined the effect of 60% normobaric oxygen on neurological activity, brain edema and hypoxia-inducible factor-1α (HIF-1α), aquaporin 4 (AQP4) and Na^+^/H^+^ exchanger 1 (NHE1) expressions in a cerebral I/R rat model [47]. 

The sham group brain structure was normal and the red-stained neurons were observed indicating neuronal death in the I/R group. Nuclear shrinkage and widespread loss of Nissl bodies in the ischemic area were noticed following cerebral ischemia-reperfusion injury. The semi-quantitative histopathological scoring was employed to quantify the H&E micrographs [48]. The symptoms were reversed by subjecting the rats to 60 or 100% normobaric oxygen. In this study, the hematoxylin and eosin staining technique was employed to evaluate the anatomical structural changes of the neuronal tissue following induction of ischemic stroke and reperfusion injury. 

**Figure 13:**
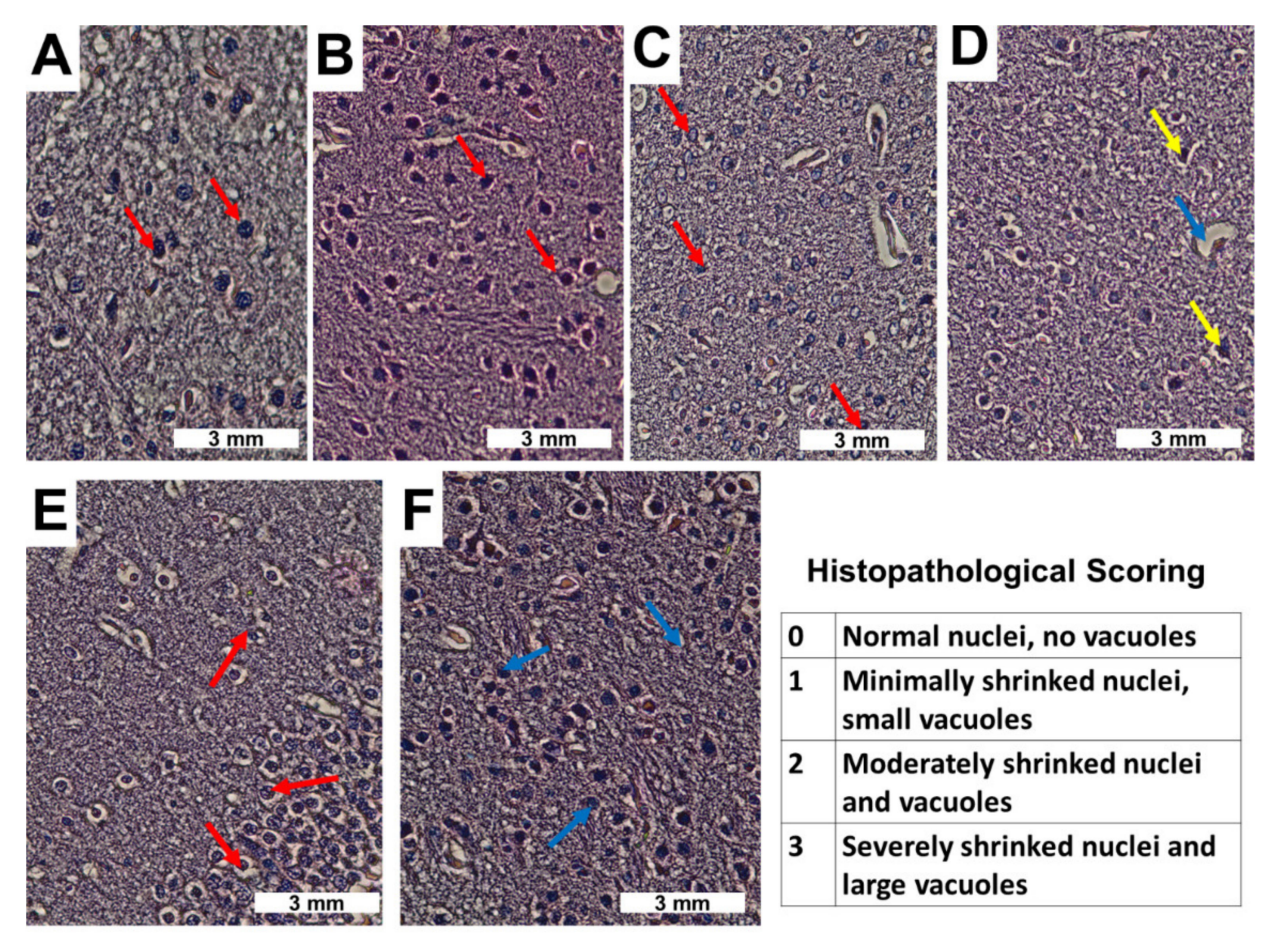
Hematoxylin and eosin (H&E) micrographs of the left and right brain hemispheres. Sham rat with right hemispheres (**A**) and left hemisphere (**B**). CCAO rat with right (**C**) and left hemispheres (**D**). FITC-DEX-PEG-ANP-CNTs treated rats with right (**E**) and left hemispheres (**F**).

There was no difference in the morphology of the neuronal tissue in the right and left hemispheres in the sham group. The staining was very intense because nuclei absorb H&E stains and become dark purple (Score 0 for normal nuclei). Nuclei were big, round, compact and clearly visible with well-defined nuclear membranes as demonstrated in Figure 13A,B (red arrows). The right hemisphere tissue was as normal as in the sham group since it was not tampered with. The nuclei in the right hemisphere of the CCAO group were normal compared to the left hemisphere nuclei (Figure 13A) (red arrows). 

The nucleolus vanished, and the nuclear membrane dissolved (Score 3 for severely shrunk nuclei). Cavitation or vacuolation was observed in the neuronal cells (Figure 13C,D) (blue and yellow arrows). Following treatment with FITC-DEX-PEG-ANP-CNTs, nuclei of the right hemisphere were intact and the morphology of the left hemisphere was immensely improved (Figure 13E,F) (red and blue arrows). The staining became intense as the nuclei absorbed the H&E stain and became dark purple (Score 2 for improved nuclei). The normal structural form of the nucleus was regained as illustrated in Figure 13E,F.

These findings have demonstrated the potential of FITC-DEX-PEG-ANP-CNTs in the reduction of inflammation and improvement of the healthy state of the neuronal tissues that underwent neurological damage and morphological changes during I/R. 

## 4. Conclusions

Vertically aligned multiwalled carbon nanotubes were synthesised with great success using Chemical Vapour Deposition. Vertical alignment was affected during the purification and functionalisation stages. They were purified, characterised, and functionalised for application in the detection and treatment of ischemic stroke. FITC, the fluorescent tag and ANP antibody, targeting ligand were successfully coupled to the DEX-PEG-CNTs. This formulation was not toxic to the rats as demonstrated by the dose-response studies in the previous work. Stroke was successfully induced using common carotid artery occlusion as demonstrated by the stroke symptoms in the rats following the occlusion procedure. ANP levels were increased following the common carotid artery occlusion and decreased after treatment with FITC-DEX-PEG-ANP-CNTs and PEG-CNTs. Fluorescence spectroscopy and microscopy confirm successful delivery of the drug and its nanocarrier to the ischemic site of the brain. These results have demonstrated the targeting ability and anti-inflammatory properties of the formulation that could induce effective treatment of ischemic stroke. This carbon nanotube system for targeting inflammatory biomarkers in ischemic stroke patients needs to be refined and further evaluated for clinical application in future. Once it has been thoroughly evaluated, it will then be further assessed in non-human primates prior to a clinical setting. 

This theranostic tool will revolutionize the diagnosis and therapy of ischemic stroke in the health industry and this may lead to the reduction of mortality rate and disability as a result of stroke hence an improvement in the global economy. These findings have demonstrated the successful development of a nanocarrier equipped with highly relevant molecules with specific functions that have the potential to be applied in delivering anti-inflammatory drugs to the diseased site in neurological diseases.

## Figures and Tables

**Table 1:** Band shifts and intensities with the degrees of functionalisation.

MWCNT Type	D Band Intensity ×10^3^	G Band Intensity ×10^3^	Intensity Increase	
			D Band	G Band
Pristine CNTs	1.18	1.63	-	-
FITC-Dex-PEG CNTs	19.60	9.81	10×	6×
Dex-PEG-CNTs	51.30	26.60	27×	16×
PEG-CNTs	92.30	47.80	49×	29×
FITC-ANP-Dex-PEG-CNTs	118	55.60	63×	34×

**Table 2:** Neurobehavioural assessment of the healthy, stroke induced, non-treated, and treated Sprague Dawley rats.

Healthy Rats	Premed	Sham	CCAO	FITC-DEX-PEG-ANP-CNTs	PEG-CNT	DEX
Healthy	Healthy	Healthy	Sick	Improved general health	Improved general health	Unhealthy
Very active and alertful	Very active and alertful	Very active and alertful	Not active	Active and alertful immediately after recovery	Active and alertful immediately after recovery	Slightly active and alertful
No stroke symptoms	No stroke symptoms	No stroke symptoms	One forelimb not fully functional	Improved gripping in both forelimbs	Improved gripping in both forelimbs	Improved gripping in both forelimbs
Circling both sides when suspended by the tail	Circling both sides when suspended by the tail	Circling both sides when suspended by the tail	Circling one side when suspended by the tail	Circling both sides when suspended by the tail	Circling both sides when suspended by the tail	Circling one side and seldom both sides when suspended by the tail
Pink eyes and of the same size	Pink eyes and of the same size	Pink eyes and of the same size	One eye dark and sunken	Improved eye (slightly reddish to pinkish colour)	Improved eye (slightly reddish to pinkish colour)	One eye affected Slightly reddish and sunken eye
-	Insignificant weight loss or unchanged weight	Insignificant loss of weight or unchanged weight	Increased weight loss compared to the premeds and shams	Insignificant loss of weight or unchanged weight	Insignificant loss of weight or unchanged weight	Insignificant loss of weight or unchanged weight

## Data Availability

Data available in Supplementary Materials.

## Supplementary Materials

The following are available online at https://www.mdpi.com/article/10.3390/pharmaceutics13091357/s1, Figure S1: Preparation of FITC labelled multiwalled carbon nanotubes. Purification by acid reflux (A). FITC labelling of PEG-MWCNTs (B), Figure S2: Spectra of the multiwalled carbon nanotubes. KBr-FTIR Spectra of the functionalised multiwalled carbon nanotubes (A). Raman Spectra of the functionalised multiwalled carbon nanotubes (B), Figure S3: Contour maps and fluorescence intensities of the FITC labelled multiwalled carbon nanotubes. FITC (A), DEX-PEG-CNTs (B), FITC-DEX-PEG-CNTs (C), FITC-DEX-PEG-ANP-CNTs (D), Fluorescence intensities of the functionalised carbon nanotubes (E-G), Figure S4: Preparation of the plasma and brain hemispheres samples. Plasma separation from the tail venous blood (A) and cardiac puncture blood (B). Preparation of brain hemispheres (C) and Figure S5: Preparation of organs and measurement of ANP levels. Organ isolation and preservation (A). Absorbance measurement of ANP (B). Natriuretic peptide mechanism of action (C).

## Abbreviations

DEX	dexamethasone
PEG	polyethylene glycol
FITC	fluorescein isothiocyanate
ANP	atrial natriuretic peptide
CNTs	carbon nanotubes
BNP	brain natriuretic peptide
VA-MWCNTs	vertically aligned multiwalled carbon nanotubes
PEG-CNTs	PEGylated carbon nanotubes
ULCCAO	unilateral common carotid artery occlusion
PEG-HCC	PEGylated hydrophilic carbon clusters
CVD	chemical vapour deposition
MCAO	middle cerebral artery occlusion
SWCNTs	single-walled carbon nanotubes
NPs	natriuretic peptides
FEEM	field excitation emission matrix
SMCC	succinimidyl-4-(*N*-maleimidomethyl) cyclohexane-1-carboxylate
BAR	Bright, Alert and Responsive
DSPE-PEG5000-amine	1,2-distearoyl-*sn*-glycero-3-phosphoethanolamine-*N*-[amino(polyethylene glycol)-5000]
