# Supplementary material for: Atrial Natriuretic Peptide Antibody-Functionalised, PEGylated Multiwalled Carbon Nanotubes for Targeted Ischemic Stroke Intervention
Source: Pharmaceutics. 2021 Aug 28;13(9):1357. doi: 10.3390/pharmaceutics13091357 (PMC8471373; doi:10.3390/pharmaceutics13091357)
Supplement: Supplementary file 1 [file pharmaceutics-13-01357-s001.zip › pharmaceutics-1298083-supplementary.pdf]

pharmaceutics

Supplementary Materials: Atrial Natriuretic Peptide Antibody-Functionalised, PEGylated Multiwalled Carbon Nanotubes for Targeted Ischemic Stroke Intervention

Patrick P. Komane, Pradeep Kumar and Yahya E. Choonara

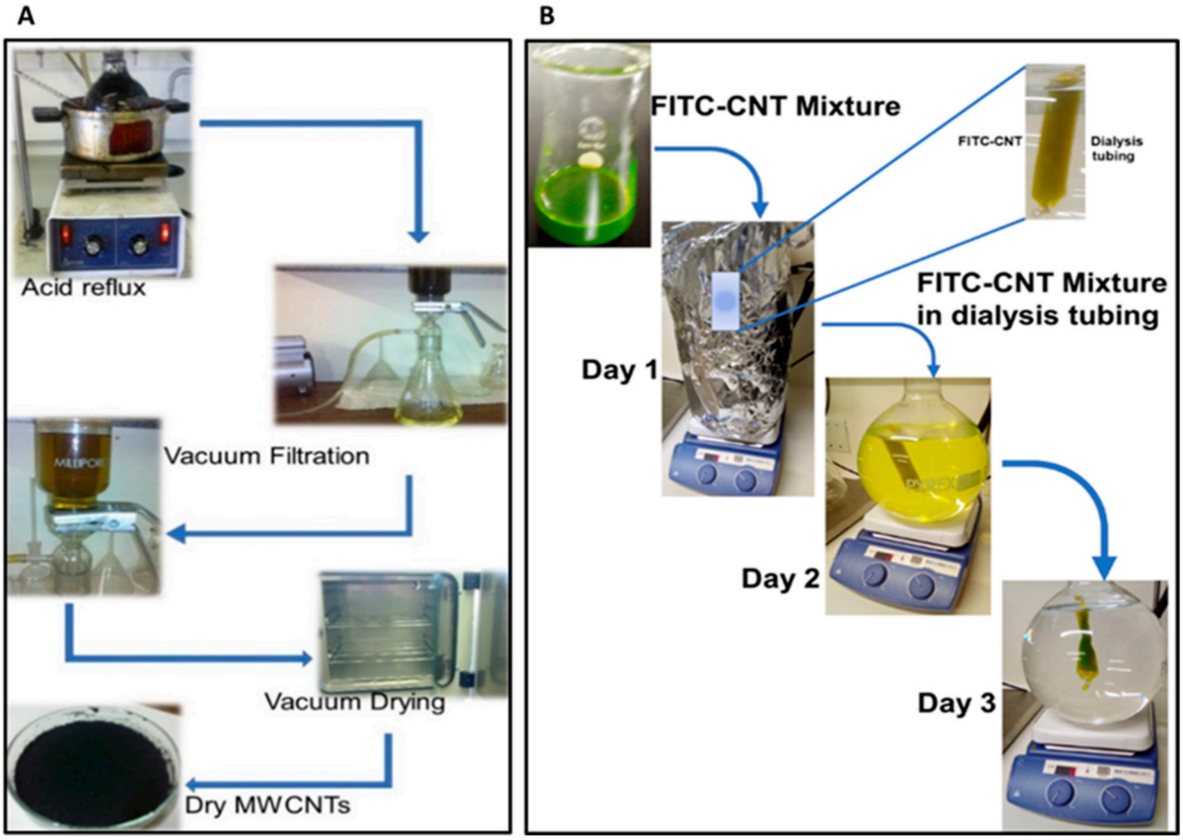

Figure S1. Preparation of FITC labelled multiwalled carbon nanotubes. Purification by acid reflux (A). FITC labelling of PEG-MWCNTs (B).

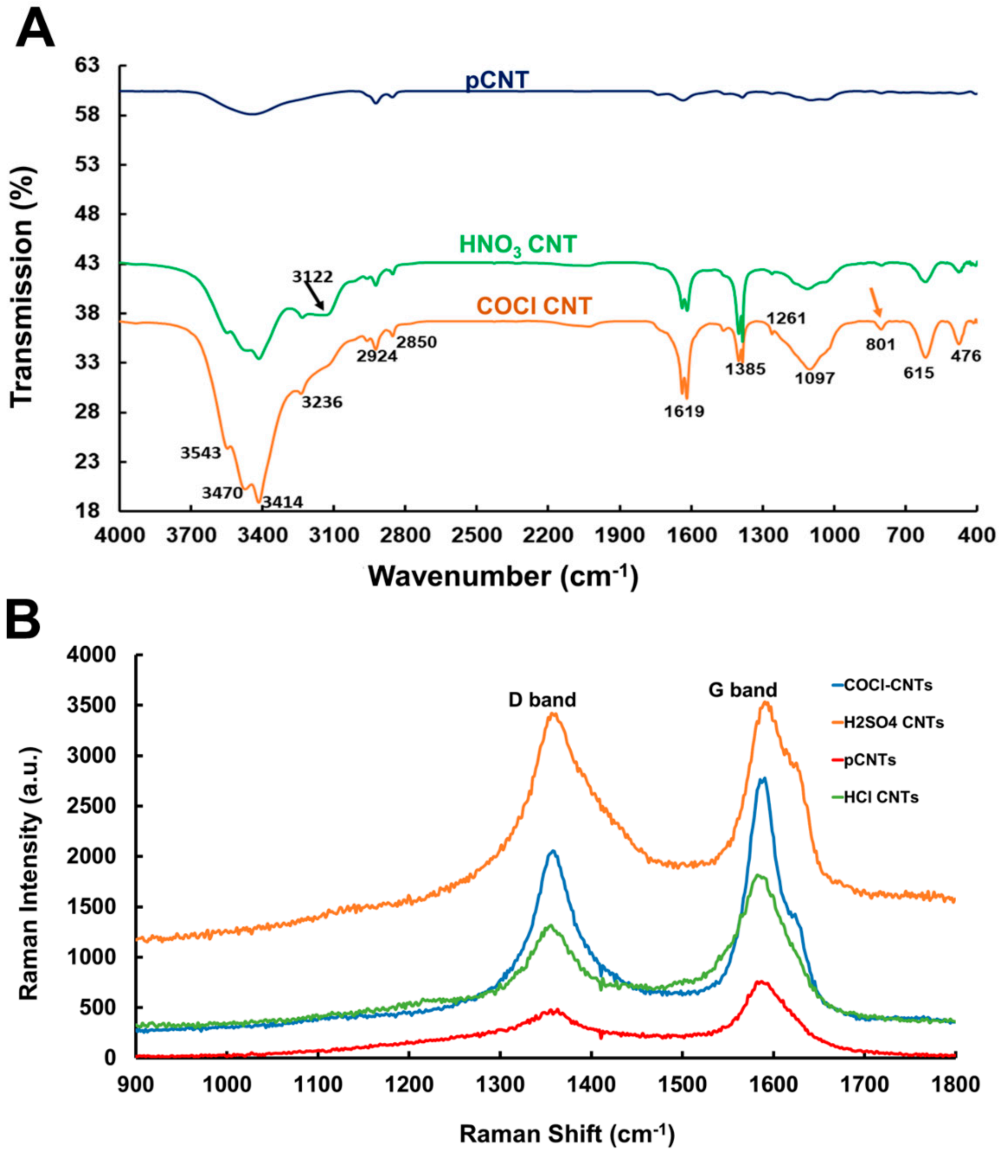

Figure S2. Spectra of the multiwalled carbon nanotubes. KBr-FITR Spectra of the functionalised multiwalled carbon nanotubes (**A**). Raman Spectra of the functionalised multiwalled carbon nanotubes (**B**).

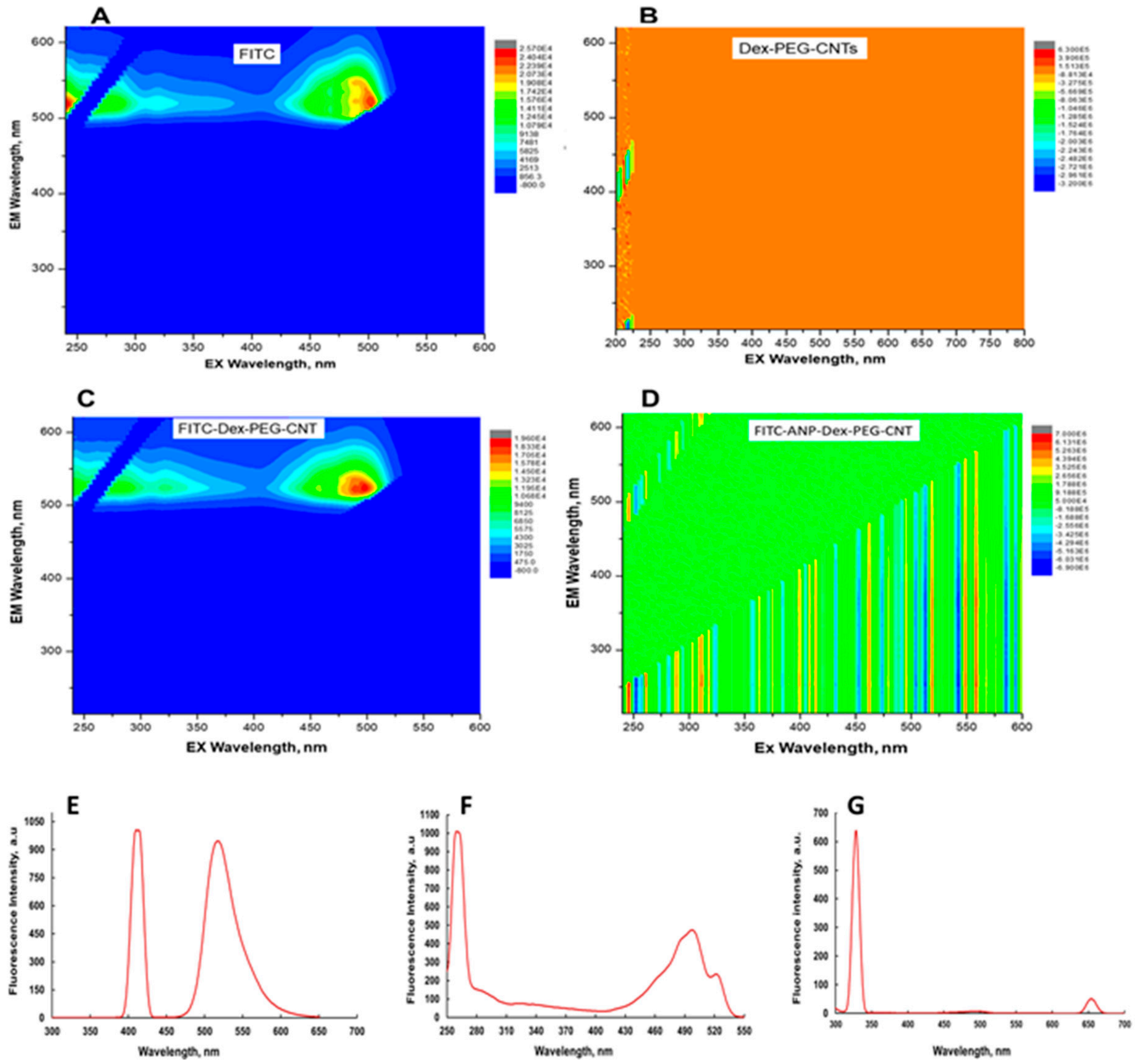

Figure S3. Contour maps and fluorescence intensities of the FITC labelled multiwalled carbon nanotubes. FITC (**A**), DEX-PEG-CNTs (**B**), FITC-DEX-PEG-CNTs (**C**), FITC-DEX-PEG-ANP-CNTs (**D**), Fluorescence intensities of the functionalised carbon nanotubes (**E**–**G**).

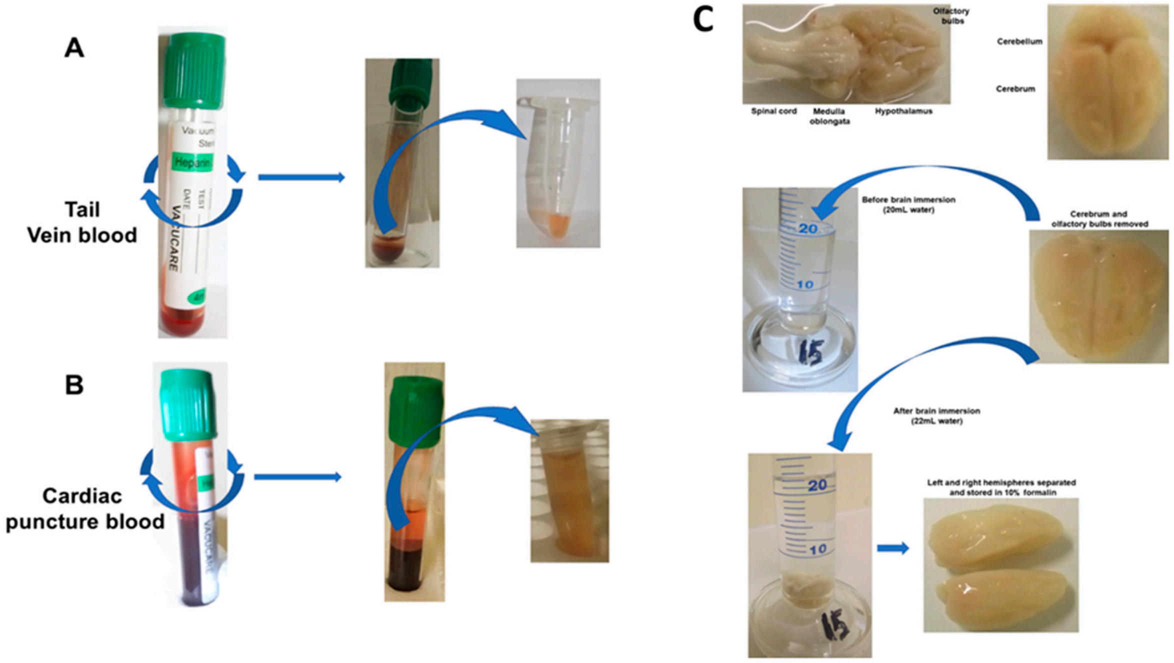

Figure S4. Preparation of the plasma and brain hemispheres samples**.** Plasma separation from the tail venous blood (**A**) and cardiac puncture blood (**B**). Preparation of brain hemispheres (**C**).

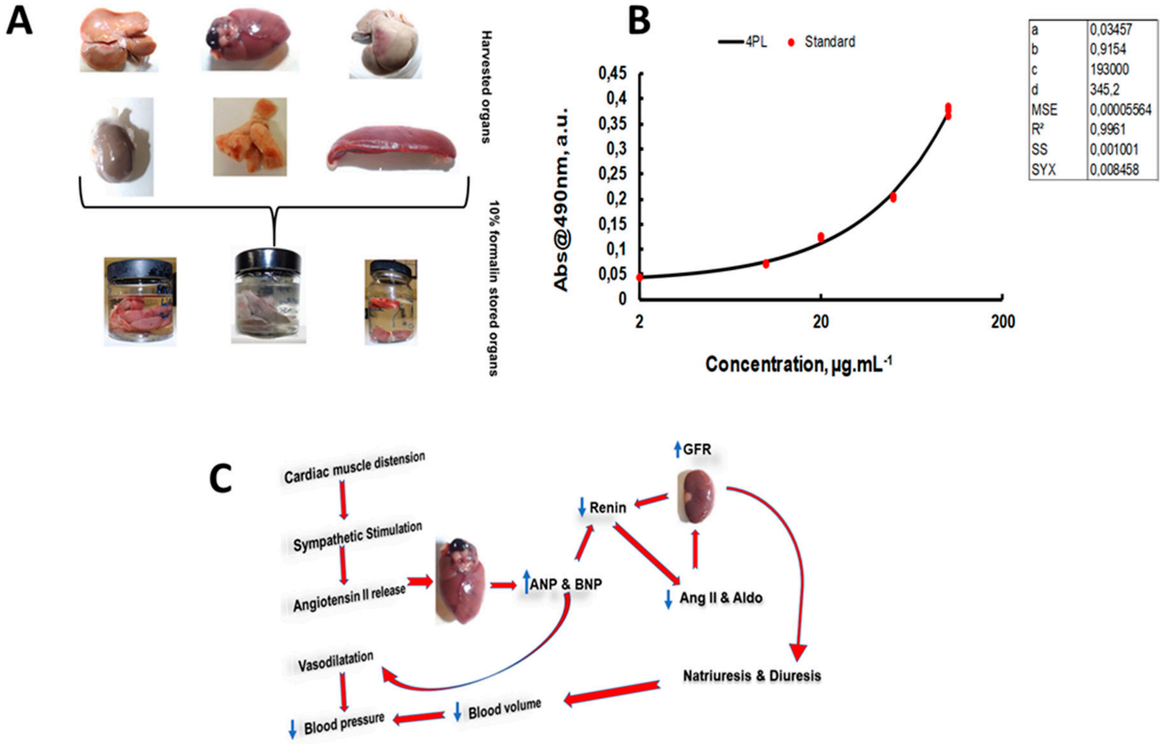

Figure S5. Preparation of organs and measurement of ANP levels. Organ isolation and preservation (**A**). Absorbance measurement of ANP (**B**). Natriuretic peptide mechanism of action (**C**).
